# Paying in public: Peer effects, impression management, and willingness to pay on digital payment platforms

**DOI:** 10.1371/journal.pone.0340550

**Published:** 2026-07-01

**Authors:** Emily A. Kiernan, Jakina Debnam Guzman

**Affiliations:** Department of Economics, Amherst College, Amherst, Massachusetts, United States of America; Gachon University, KOREA, REPUBLIC OF

## Abstract

How do the social features of payment forms shape willingness to pay (WTP)? We conduct a purchasing experiment during Covid-19 in which 261 U.S. undergraduates are randomly assigned a payment form in which to complete a real transaction – either via debit card, credit card, or via one of the payment visibility settings within *Venmo*. We then elicit both stated preferences and WTP for ten household items. We find that the previously documented digital payment premium only persists when transactions are either private or are visible to the public at-large. When transactions are visible only to participants’ friends, however, we find a large *decrease* in their WTP consistent with impression management when peers are frugal. We find that viewing others’ transactions increases the magnitude of this effect. Payment visibility matters for WTP, but causes no differences in stated preference. These findings highlight the importance of considering social features of payment forms in addition to more well-documented features such as convenience, transparency and pain of payment.

## Introduction

Payment mechanism – whether cash, credit, debit, mobile or digital payment – has been shown to matter for purchase satisfaction [1], brand loyalty [2], and willingness-to-pay (WTP) [3–7]. As potential moderators driving these differences, the literature has centered on differences across payment methods in convenience [5,8], tangibility [4,9–11], and pain of payment [11–13]. These mechanisms, however, neglect a key aspect of many mobile and digital payments – components of these payment forms are social. Purchasing behavior may be visible to others beyond the two transacting parties, with transactions embodying communication as well as an exchange of money and goods. For example, some payment platforms include social feeds which constantly update users about the purchasing behaviors of others. In these platforms, consumers may also choose a more, or less, socially distant social group with which to share their purchases. Design features of digital payment platforms can bring the *felt presence* of others into purchasing decisions.

We therefore consider the role that impression management behaviors– or behaviors which stem from individuals’ motives to maintain a desired image in the presence of others [14] – play in determining WTP when using digital payments. Some related findings in the physical retail space include impression management motives for: increased spending for agency-oriented consumers and decreased spending for communion-oriented consumers in the presence of others [15]; decreased spending in the presence of an attractive salesperson [16]; and foregoing coupons to avoid looking cheap in front of others [17]. While the majority of work examining the role of impression management in retail has considered a physically present other or set of others, impression management has been found to matter in the felt presence of others in a variety of digital environments (for a recent overview of findings, see Blunden and Brodsky [18]). In this context, we consider the possibility that consumers may engage in impression management in response to the felt presence of others when determining their WTP using a digital payment method.

During Covid-19, we conducted an experiment among undergraduate students, a population for which impression management concerns are particularly important [19]. We conduct a real-stakes experiment in which we offer 261 college-student participants living in the United States the opportunity to purchase everyday items and randomly vary the audience to which participants’ digital payment transactions are visible – experimental transactions are either private, publicly visible to all platform users, or visible only to users’ friends. Public consumption contexts produce stronger impression management than private ones [20–21], and known audiences produce stronger impression management than unknown audiences [14]. We therefore expect that, relative to private transactions, impression management concerns will be higher among transactions which are publicly visible; we expect that impression management concerns will be yet higher still when transactions are visible only to users’ friends.

As the everyday items we use are unlikely to signal identity, we do not anticipate that impression management motivations will, *a priori*, increase the WTP of any individual or subgroup for these items. Instead, we expect that, consistent with the work of Jia, Liu and Lowry [22], impression management concerns will lead to behaviors biased toward the norm prevalent within the group to which digital payments are visible. We demonstrate that in a time of unprecedented economic hardship among U.S. college students, college students *decreased* their WTP. Consistent with impression management, we find that, relative to private transactions, transactions which are visible to a user’s friends are statistically significantly lower by $0.44 (s.e. = 0.26).

## Research framework

### Impression management in virtual transactions

Impression management is “the process by which individuals attempt to control the impressions others form of them” (Leary and Kowalski p. 34 [14]). In some settings, people are motivated to control the way in which others perceive them. Leary and Kowalski decompose impression management into two separate processes. The first is *impression motivation*, or the desire to create a perception of oneself. Impression motivation is higher when impression management behaviors will solidify an identity, maintain or enhance self-esteem, improve material outcomes, or, as is relevant in our case, improve social outcomes such as approval or friendship [14]. Leary and Kowalski’s second process of impression management is *impression construction,* or the way people choose a particular perception and choose actions to bolster that desired perception. Observed impression construction behaviors include, among a panoply of others, changes in WTP [20] and in purchasing behavior [15–17]. Individuals may use purchasing decisions to influence the attributions others make of them.

Unlike Goffman’s idea of self-presentation which may involve a performance of images (or “impressions”) that are only self-relevant, impression management is inherently other-regarding [23]. Individuals, therefore, engage in more impression management when behaviors are public rather than private [14]. For example, Berger experimentally elicits consumers’ WTP for environmentally responsible products and finds that consumers are willing to pay more for these products when this choice is made public [21]. Griskevicius et al. find that men, after being shown attractive photos, spend more on publicly consumed purchases than on privately consumed ones [20].

Critically, beyond the simple distinction between public and private behaviors, the strength of the impression management motivation depends on the characteristics of the audience. Specifically, impression motivation is greater when an individual has had more contact with the other, when the individual expects to have future interactions with the other, and when the other holds sway over the individual's future (material or social) outcomes [14] – all of these characteristics can be found in friendship relationships. Impression management motivations should be heightened in the presence of an individual’s friends.

Indeed, in their 2020 review of work considering the role of others on consumer behavior, Argo and Dahl highlight the centrality of impression management as a driver of consumer behavior in the presence of friends [24]. For example, in a series of real shopping settings, Kurt, Inman and Argo [15] hypothesize that concerns about impression management may dictate the effect of shopping with a friend on own purchasing behavior. They find that when consumers display high levels of “agency”, that is, when they are motivated to promote the self rather than others, consumers spend more when shopping with a friend than when alone. Consumers who do not have high levels of agency, however, spend less when shopping with a friend. While there has been much more limited discussion of impression management in virtual retail settings, a notable study is work by Jia, Liu and Lowry [22], in which researchers find that when peer networks are visible, consumer purchases conform to the online purchasing decisions of peers to gain social approval. When transactions are made private, the role of normative social influence disappears. These results are consistent with impression management behavior, though the authors do not explicitly interpret their findings through this lens.

The shape which impression management behaviors take depends on their target audience. Below we argue that during Covid-19, at the time of the experiment, the felt presence of friends may have activated a desire to convey a perception of frugality, being appropriately modest in one's spending during a time of widespread economic distress. In the context of Covid-19, we suggest that impression management concerns decreased WTP.

### Financial hardship during Covid-19

The period in which our study was conducted was one of unprecedented economic disruption and severe financial hardship. Analyses using contemporaneous credit card and consumption data show substantial declines in spending in the United States during the early phase of Covid-19 with estimates of the aggregate decline ranging from −27.8% to −40% [25–27]. College students were not insulated from the economic disruption. A 2020 survey of City University of New York students found that 81.1% of students reported that a member of their household had lost income due to the pandemic [28]. In their study focusing on ethnically diverse college students, Molock and Parchem found that 54% of students reported a disruptive change in their personal finances [29]. In their survey of 1,500 students at a large public institution in the United States, Aucejo, French, Araya and Zafar found that working students’ wages decreased (by 31%) and that their hours worked decreased as well (by 37%). Students reported losing internships, jobs and job offers, while 61% reported a decrease in their household income [30]. We therefore suggest that, during the time of our study, frugality was the dominant norm regarding spending among college-aged peer groups. We suggest that impression management operated as undergraduate students “tailor[ed] their public images to the perceived values and preferences of significant others” (Leary and Kowalski, p. 41) in the presence of this frugality norm.

### Mobile as a payment form

There is a long literature exploring the importance of payment form for willingness to pay. In early work, Prelec and Simester use an experiment involving real transactions to find an increase of up to 100% in willingness-to-pay when participants use credit cards rather than cash [6]. More recent work has considered mobile payments in addition to cash, credit and debit payment methods. In a real-stakes purchasing experiment, Falk, Kunz, Schepers, and Mrozek find a “mobile premium” for items purchased using mobile payments relative to cash, though they find no difference between mobile and credit valuations [3]. Boden, Maier, and Wilken also find that mobile payments are associated with higher WTP in a series of hypothetical shopping experiments [8]. Importantly, these studies consider mobile payments in general rather than within any particular mobile payment platform.

A prominent idea to resolve valuation gaps across payment forms is that *pain of payment* differs across forms. Pain of payment posits that consumers experience a negative affective reaction when parting with money [31]. Since credit cards, contactless payments and mobile payments decouple the decision to spend from the actual transfer of funds (and the pain associated with this transfer), consumers are willing to pay more for transactions made using these methods than for those made using cash [12]. Pain of payment may also increase with the transparency of a transaction. Prelec and Simester hypothesize that the higher willingness to pay with credit cards they observe is due to the lack of transparency associated with this payment form [6]. Falk et. al [3] find that mobile payments are even less transparent than credit card payments (which, in turn, are less transparent than cash payments). Important for our purposes, pain of payment may also decrease the more routinely (habitually) a particular payment form is used. Broekhoff and van der Cuijsen provide evidence that the pain of payment decreases as use of a payment form becomes more routine and automatic. When examining contactless payments in Denmark, they find that individuals who use contactless payments more intensively have less pain of payment when using this payment method [32].

Finally, increased convenience may lead to increases in consumers’ WTP [5]. Mobile payments are contactless and require neither a pin nor a wallet; their convenience is one of the most important reasons consumers choose to adopt their use [33]. Among users who have fully adopted mobile payment forms and use them routinely, we may expect that the convenience of using these forms is greater still. Boden et al. examine this mechanism directly and find empirical evidence that the convenience of mobile payments drives increased spending relative to credit cards and cash [8]. Our experiment contributes to this work by considering both the mobile nature of payments as payment forms as well as the social nature of platforms such as Zelle, Venmo and CashApp. We also consider whether our treatment effects differ among frequent Venmo users for whom the pain of payment may be lower.

### Venmo

Venmo is a digital payment platform that allows users in the United States to send and receive payments to and from other Venmo users and select retailers. As of the second quarter of 2022, Venmo had nearly 90 million active accounts [34]. Over time, Venmo has been growing in total payment volume; from 2018 to 2021 volume increased by 8% from $19 billion to $58 billion [35,36]. Further, Venmo is also growing in user engagement, as average transaction frequency tripled from 2012 to 2016 [37]. Young people are much more likely to use digital payments and Venmo specifically. A 2022 survey found that 57% of 18- to 29-year-olds use Venmo while only 15% of those 65 and older have [37]. Venmo reports that 35% of Venmo users are 18–29, compared to 23% of the overall US population [38]. In 2023, Venmo processed an estimated $270 billion dollars in total payment volume [39] and PayPal, the parent company of Venmo, projects total payment volume to grow at a 20% compounded annual growth rate from 2024 to 2027 [40].

A Venmo transaction requires the initiator to indicate the user they want to send to or request from, enter the dollar amount of the transaction, and write a caption for the payment. All money received is added to a user’s Venmo balance, which can then be transferred to a bank account. To send money, one can use their Venmo balance or link a bank account, debit card, or credit card. Venmo is generally free to use; fees are only incurred when using a credit card (3% of the transaction amount) or when initiating an instant bank transfer. If Venmo users could not transfer money out of their accounts, then it would be rational for them to treat their Venmo balance differently because it would not be a perfect substitute for cash or credit. However, money in a user’s Venmo balance is easy to transfer directly to their bank account; users can choose between an instant transfer for a 1.75% fee (minimum of $0.25; capped at $25) or a free transfer that takes 1–3 business days. While it is becoming more common for retailers to accept Venmo, most Venmo transactions are peer-to-peer transfers. Zhang et al. find that the majority of Venmo transactions fall into the categories of food/drinks or transportation, with less popular categories being utilities and entertainment [41].

Venmo differs from other digital payment platforms like PayPal and Zelle as it includes a social media feed or a “social awareness stream” (SAS) in which users see a stream of other users’ transactions. The default setting for payment visibility is *Public*, but users can change their privacy setting for all future transactions or for one individual transaction. *Public* transactions “will be shared on the Venmo public feed and anyone on the internet will be able to see it” and *Friends* transactions “will only be shared with your Venmo friends and with the other participant’s Venmo friends” [42]. Venmo users are generally inclined to add friends on the app; half of Venmo users have at least 40 friends and 30% have over 100 friends [41]. For *Private* transactions, however, Venmo “will not share the transaction anywhere other than your own personal feed and, if it's a payment to another user, the feed of the other person in the payment” [42]. As of 2016, these *Private* transactions made up approximately 51% of all Venmo transactions since Venmo became public to users in 2012 [41]. While Venmo users describe themselves as indifferent or neutral towards the social feed in surveys, they report putting a lot of thought into the captions of their transactions on Venmo [43]. Others have looked at Venmo as social media [44,45], finding that users use that platform for its social benefits as well as for its purely task-driven use [43].

## Research design

The Amherst College Institutional Review Board (IRB) approved the study (Protocol #20–041) prior to the experiment, and written informed consent was obtained for each participant. Screenshots from the survey, including the text used for informed consent, are available in S1 Fig. To understand the role of social factors and payment methods on consumer WTP, we randomly assigned participants to one of five payment methods and then asked participants to participate in ten real-stakes Becker, DeGroot, and Marschak (BDM) lotteries for household goods. This research design is similar to that of Runnemark, Hedman, and Xiao which was conducted among masters students in Denmark [46], in which they used a BDM mechanism to elicit WTP for beer or coffee after randomly assigning the students to complete their subsequent payment using either debit cards or cash. In our experiment, we consider in addition to debit card transactions, credit and digital (Venmo) transactions. Notably, three of the assigned payment methods varied the experience of social features within the Venmo platform. We use this variation to understand the role of peer effects on participants’ willingness to pay in addition to the effects of the platforms themselves.

We conducted an online experiment during January and February of 2021 with 261 undergraduates attending fifteen colleges or universities across the Northeastern United States. Participants were recruited through a link distributed by faculty at their institutions and informed consent was elicited from participants prior to the start of the experiment. Each participant was compensated with a $5 Amazon gift card upon completion of the experiment.

Prior to the experiment, prospective participants answered a series of screening questions. Qualifying participants were required to: 1) have access to either a debit or credit card, 2) be 18 years of age or older, and 3) be an active Venmo user. To qualify as a Venmo user, each participant needed to have a Venmo account, have the Venmo app downloaded, and have either a nonzero Venmo balance or an alternate payment form linked to their Venmo account.

Qualifying participants were then randomly assigned to one of five payment form treatment groups: 1) debit card, 2) credit card, 3) Venmo-*Private* (in which user transactions are only visible to the transacting dyad), 4) Venmo-*Friends* (in which user transactions are visible, not only to the transacting dyad, but also to the “friends” of the transacting dyad), and 5) Venmo-*Public* (in which user transactions are visible to all Venmo users). Participants were then told that they can only use their assigned form of payment for the rest of the experiment, and then asked to affirm this. Venmo treatment groups were asked to open the app and change their privacy settings to their randomly assigned setting (*Public*, *Friends*, or *Private*), with detailed instructions provided to implement the change. Additionally, for participants in any of the three Venmo payment groups, there was a sub-treatment: transactional priming. Within each Venmo group, half of participants were randomly selected and asked to browse through their *Public* Venmo social awareness streams for 2 minutes (unassigned participants simply proceeded with the experiment). While we are underpowered to identify statistically significant effects in this sub-treatment arm, we include it to explore the effect of making a transaction’s audience more salient to participants.

The experiment consisted of ten incentive-compatible BDM lotteries for ten different low-cost household goods: a notebook, a pen, an adhesive phone wallet, a can of soda, a pack of gum, a granola bar, a bottle of coffee, a bar of soap, a tube of toothpaste, and a cloth face mask. In order to avoid any “winner” or “loser” effects, participants listed their maximum price for all ten items before drawing any prices; essentially, we ran ten BDM lotteries simultaneously (rather than back-to-back, as in this case the previous lottery outcomes could have influenced the subsequent WTP estimates). Thus, only after the participants provided their WTP for all ten items was a random price between $1.00 and $5.00 drawn for each good. Participants were then shown all the random prices followed by whether they won (i.e., if the randomly drawn price was lower than or equal to their WTP) or lost each lottery. S1 Fig demonstrates how lottery results were communicated.

Those who did not win any lotteries were not given the opportunity to transact and thus proceeded to final demographic questions. Those who won one lottery were asked to purchase the item they won at the randomly drawn price via their assigned payment form. Winners of multiple lotteries had one winning item chosen at random and were asked to purchase this winning item. After the random selection, they paid for their item at the randomly drawn price via their assigned payment form, just like those who won only one lottery. All transactions are secure for participants, and participants were able to opt-out of making a payment (though only 1.6% of participants opt-out of payments). Details about payment processing are available in S1 Fig.

After the payment phase (or, in the case that the participant had zero wins, directly after the lotteries), participants answered a series of follow-up questions about their demographics, typical spending habits, payment preferences, Venmo usage and social media usage. Lastly, participants were debriefed. After completing the experiment, each participant received a $5 Amazon e-gift card via email. If they purchased an item during the experiment, it was mailed to them the following business day.

### Pilot study

Prior to running the full experiment, we executed a pilot on Amazon mTurk on January 5, 2021 with 49 participants to assess levels of attrition and payment compliance. Of 49 participants, only 25 correctly answered all screening questions and won at least one lottery. Among the 25 which reported paying for a winning item, only one participant actually submitted a payment. Given the data quality, we modified our recruitment procedure prior to running the full experiment. First, we decided to recruit college students in the Northeastern United States directly through their institutions rather than through mTurk. This recruitment method was chosen to increase participant confidence in the experiment and in making a real-stakes purchasing decision during an online experiment. Second, we added more attention checks and verification questions to understand participant motives for non-payment following a winning lottery.

## Results

Summary statistics are presented in Table 1 (The complete replication package is available at https://github.com/jakina/venmo-experiment). After removing participants who failed to complete the experiment or answered all attention check questions incorrectly, a total of 261 participants completed the experiment. Data cleaning involved removing 26 observations from participants who either: failed to complete a payment (6 participants), completed the survey twice (1 participant), reported having no willingness to pay for any of the items (11 participants), or who provided contradictory personal information throughout the course of the experiment (e.g., participants who reported that they both do, and do not own a debit card) (8 participants). After cleaning, we are left with 2,350 valuation decisions from 235 unique participants. For the interested reader, we replicate all estimations contained in the manuscript for the full intent-to-treat sample in S2-S4 Tables. These tables show that coefficient estimates and statistical significance change little by considering the intent-to-treat, rather than the cleaned, sample. Average willingness to pay for each household item is uniformly lower than the market value for each item and varies widely across items: notebook (mean = $1.21; s.e. = 0.096), pen (mean = $0.71; s.e. = 0.57), adhesive phone wallet ($1.18; s.e. = 0.12), can of soda (mean = $0.57; s.e. = 0.048), pack of gum (mean = $0.94; s.e. = 0.054), granola bar (mean = $0.45; s.e. = 0.04), bottle of coffee (mean = $1.48; s.e. = 0.10), bar of soap (mean = $1.05; s.e. = 0.083), tube of toothpaste (mean = $1.68; s.e. = 0.099), and cloth face mask (mean = $2.04; s.e. = 0.44). The mean WTP across all items and treatment arms is $1.13 (s.e. = 0.051) and the median is $0.50. Table 1 shows mean values of selected summary statistics across the five main treatment groups. For all characteristics we fail to reject the null hypothesis of mean equality across all treatment groups. With three exceptions this holds for pairwise tests across treatment groups as well. For the complete set of pairwise t-tests, see S5 Table.

**Table 1:** Summary of demographic and Venmo usage statistics.

	(1)	(2)	(3)	(4)	(5)	(6)	
	Total	Credit	Debit	Venmo-*Private*	Venmo-*Friends*	Venmo-*Public*	
	N = 235	N = 33	N = 50	N = 49	N = 53	N = 50	
	Mean/(SE)	Mean/(SE)	Mean/(SE)	Mean/(SE)	Mean/(SE)	Mean/(SE)	F-stat/(P-value)
Age	20.162	20.424	20.180	20.082	19.981	20.240	0.735
	(0.082)	(0.238)	(0.166)	(0.162)	(0.184)	(0.182)	(0.569)
Year of Study	2.638	2.758	2.720	2.510	2.509	2.740	0.614
	(0.072)	(0.180)	(0.149)	(0.157)	(0.167)	(0.153)	(0.653)
Female	0.626	0.545	0.700	0.592	0.717	0.540	1.450
	(0.032)	(0.088)	(0.065)	(0.071)	(0.062)	(0.071)	(0.219)
White	0.689	0.818	0.640	0.653	0.679	0.700	0.864
	(0.030)	(0.068)	(0.069)	(0.069)	(0.065)	(0.065)	(0.486)
Household Income	3.863	3.759	4.000	3.478	4.068	4.000	0.978
	(0.115)	(0.332)	(0.229)	(0.265)	(0.242)	(0.237)	(0.421)
Venmo Balance	97.208	147.302	83.029	84.907	94.026	93.753	0.293
	(19.080)	(98.291)	(23.943)	(24.919)	(39.949)	(31.639)	(0.883)
Years with Venmo Account	2.952	2.970	3.080	3.021	2.804	2.898	0.334
	(0.086)	(0.236)	(0.173)	(0.189)	(0.179)	(0.203)	(0.855)
Friends on Venmo App	100.553	104.182	96.640	96.082	97.868	109.300	0.216
	(5.571)	(17.282)	(10.882)	(10.795)	(12.953)	(12.089)	(0.929)
Venmo Transactions/Mo.	3.278	4.970	2.718	2.500	3.698	3.040	1.429
	(0.338)	(2.062)	(0.305)	(0.275)	(0.551)	(0.394)	(0.225)
Venmo Spending Percentage	16.232	12.970	14.651	16.449	19.472	16.320	1.186
	(0.965)	(1.476)	(2.031)	(2.115)	(2.204)	(2.380)	(0.318)
Number of Social Sites Used	5.000	5.303	5.300	5.122	4.731	4.646	1.178
	(0.129)	(0.321)	(0.262)	(0.297)	(0.282)	(0.278)	(0.321)
Hours on Social Media/Day	2.908	2.864	2.925	2.929	2.713	3.105	0.299
	(0.119)	(0.471)	(0.242)	(0.280)	(0.174)	(0.234)	(0.879)

This table reports the mean values for selected demographic characteristics and Venmo usage statistics across the five treatment and control groups in the experiment. Standard errors are given in parentheses. Household income has been recoded as the mean value of the categorical income range reported by participants. Included participants include the 235 participants who completed payments using their randomly assigned payment forms and are included in the analyses.

Relative to the average college student in the United States, women and high-income students are overrepresented in our sample. The gender composition of the sample is 62.6% female, 35.3% male, 2.1% nonbinary. A majority of participants have a household income greater than $100,000, while the remaining participants are roughly evenly distributed across the remaining household income categories. Participants represent all undergraduate years of study, with the average year of study being roughly between a sophomore and junior. The average participant is approximately 20 years old. 68.9% of participants indicated that they identify as White, compared to 29.8% identifying as Asian, 3.4% identifying as Black, and 0.4% identifying as American Indian or Alaska Native. Only 7.2% of all participants indicated that they are of Hispanic, Latino, or Spanish origin.

The average participant in our experiment is an active Venmo user but uses credit or debit cards as their primary payment method. At the time of the experiment, participants had an average of 100.55 friends on the Venmo app and $97.21 in their Venmo balance (though this is broadly distributed with a median of $11). Participants use Venmo for an average of 3.3 transactions per month and self-report spending $23 and receiving $27 on Venmo in an average month. Venmo transactions account for 16% of participants’ total spending, which is less than is accounted for by credit or debit transactions but more than cash or other digital payment platforms. Participants use an average of 1.4 other digital payment platforms in addition to Venmo, the most popular of which is PayPal. For financial transactions, the average participant most uses a credit card, followed by debit, Venmo, cash, and any other digital payment platform, in that order.

### Main effects

**Fig 1:**
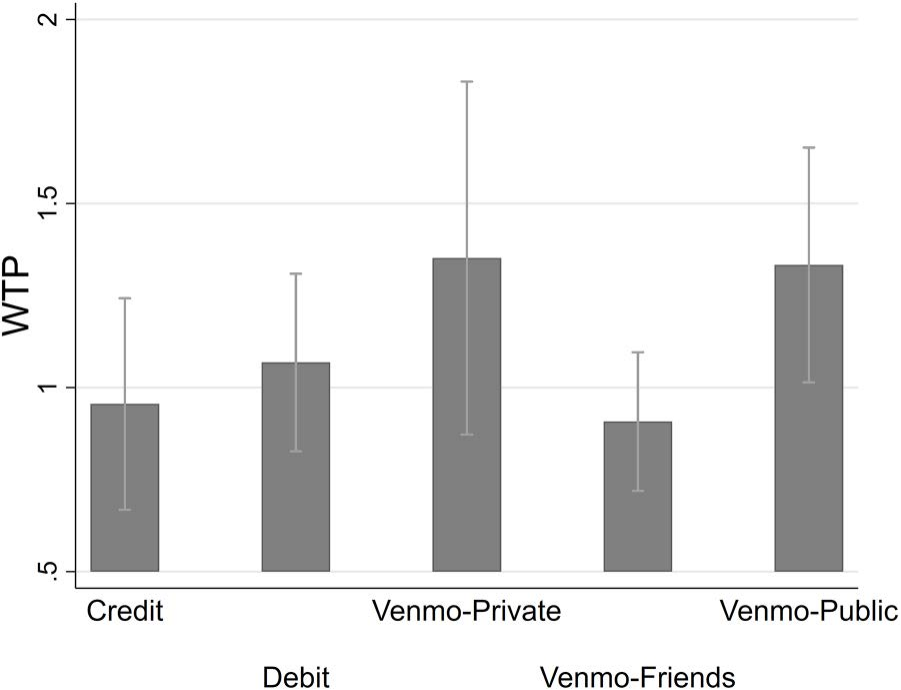
Mean WTP for each treatment arm (in dollars) with 95% confidence intervals. This figure illustrates mean willingness to pay across all items for each treatment group. 95% confidence intervals are shown with standard errors clustered at the participant level.

Fig 1 shows mean WTP with 95% confidence intervals for the average item in each of the five main treatment arms, where standard errors have been clustered at the participant level: credit ($0.95; s.d. = 1.34), debit ($1.06; s.d. = 1.31), Venmo-*Private* ($1.35; s.d. = 4.69), Venmo-*Friends* ($0.91; s.d. = 1.20), and Venmo-*Public* ($1.33; s.d. = 1.58). Consistent with previous work examining willingness to pay in digital payment platforms [8], our point estimates suggest that participants assigned to Venmo-*Public* and *Private* payment arms had higher WTP on average than did those in credit or debit arms. We can reject at a reasonable level of statistical significance the null hypothesis that WTP in these two Venmo groups is equal to that within the credit and debit treatments (*F*(1, 180) = 3.38; *p* = 0.068). Participants in Venmo treatment where transactions are visible only to the sender and recipient (Venmo*-Private*) have the highest average willingness to pay, $0.40 greater than participants assigned to the credit group, and $0.28 greater than participants in the debit group without controls. Inconsistent with previous work comparing mobile payments with credit cards [3,8], however, we find that WTP in the Venmo-*Friends* condition is lower than that in both the debit and credit groups, though this difference is not statistically significant (*F*(1,135) = 0.74; *p* = 0.39). These point estimates are consistent with impression management behaviors in the felt presence of frugal peers – we see that WTP is lower in the Venmo-*Friends* condition than in the Venmo-*Private* condition. With exception of items related to Covid-19, when disaggregated by item type, these patterns hold (see S6-S9 Tables). As is shown in S10 Fig, mean stated preference ratings are quite similar across all treatments, suggesting that there was no effect of payment form on participant preferences for the items.

To examine the role of impression management on valuations and item preference, we estimate the following ordinary least squares regression:


$$\begin{array}{c} \overset{k}{\underset{i,j}{Y}}=\alpha_{\text{0}}+PaymentFor\overset{\prime }{\underset{i}{m}}\alpha_{1}+\alpha_{\text{2}}Item_{j}+\varepsilon_{i,j} \end{array}$$ (1)

where *i* represents an individual respondent and *j* represents the lottery item, $\overset{k}{\underset{i,j}{Y}}$ is the dependent variable of interest – alternatively individual *i*’s WTP for item *j* or reported preference rating for item *j.* The vector of variables *PaymentForm*_*i*_ indicate the payment form to which the participant was randomly assigned to complete the experimental payment. Finally, due to the systematic differences in item-level means described above, we include item-level fixed effects in all specifications (*Item*_*j*_). Regression results are reported in Table 2 for WTP, and S11 Table for product ratings. In columns (2) – (4) we add to equation one additional control variables, either demographic characteristics (participant age, race, gender, and income), Venmo usage measures (the number of friends that a participant has associated with their Venmo account, the age of the Venmo account, average monthly Venmo inflows and outflows, the percentage of their monthly spending which is done using Venmo, the number of Venmo transactions a participant completes in a month, and the number of payment methods which a participant has chosen to link to their Venmo account), or both. In all specifications, standard errors are clustered at the participant level.

**Table 2:** Estimates from regression of WTP on payment form.

DV: *WTP*	(1)	(2)	(3)	(4)
Debit Card	−0.284 (0.272)	−0.253 (0.255)	−0.378 (0.305)	−0.343 (0.289)
Credit Card	−0.397 (0.283)	−0.359 (0.263)	−0.373 (0.297)	−0.324 (0.281)
Venmo – Friends	−0.444^*^ (0.261)	−0.438^*^ (0.263)	−0.545^*^ (0.292)	−0.502^*^ (0.293)
Venmo – Public	−0.019 (0.292)	−0.065 (0.268)	−0.054 (0.318)	−0.113 (0.308)
Demographic Controls	N	N	Y	Y
Venmo Usage Controls	N	Y	N	Y
Item FE	Y	Y	Y	Y
Constant	1.437^***^	1.543^***^	2.499^*^	1.939
	(0.224)	(0.302)	(1.388)	(1.415)
Observations	2,340	2,290	2,040	2,000
R-Squared	0.043	0.048	0.047	0.051

This table reports coefficient estimates from estimating Equation 1 where the dependent variable of interest is an individual’s WTP. Estimates are reported with and without demographic and Venmo usage controls. All specifications include item-level fixed effects. Venmo-Private is the omitted category in all specifications. Robust standard errors, clustered at the participant level, are reported in parentheses. *** p < 0.01, ** p < 0.05, * p < 0.1.

We note that the coefficient estimates of the effect of being assigned to the Venmo-*Friends* treatment change little with and without the inclusion of controls. We do however, gain statistical power given our relatively small sample size and precision in the estimates by doing so. As shown in Table 1, average values of the Venmo usage variables are quite different across the three Venmo treatment conditions (though not statistically distinct at conventional levels). We therefore include these usage controls in our preferred specification as they are likely to predict a user’s convenience and pain of payment, and therefore WTP.

In Table 2 we find evidence consistent with impression management behavior in the presence of a frugality norm. Across all specifications, we find that relative to participants in the Venmo*-Private* treatment arm, participants are willing to pay statistically significantly less (between $0.44 (*p* = 0.091) and $0.55 (*p* = 0.064)) for the average item in the Venmo-*Friends* treatment. Given that the mean WTP across all items in the Venmo-*Friends* group is $0.91 (s.e. = 0.052), a decrease of $0.44 represents a 48.5% decrease in WTP. While not statistically significantly distinct (recall that there are between 33 and 53 participants per treatment group), point estimates suggest that perhaps, relative to Venmo-*Private*, WTP may have been lower in the Venmo-*Public* treatment. This is directionally consistent with impression management in the presence of a less well-known audience. Across all payment forms, point estimates suggest that WTP is the highest when Venmo is used for a private transaction (depending on the specification, mean WTP in the debit treatment is about $0.34 lower (s.e. = 0.29) and mean WTP in the credit treatment is about $0.32 lower (s.e. = 0.28)). Taken together, these results suggest a nuanced role of payment visibility for willingness to pay – a private use of a digital payments increases WTP, while anticipating the digital gaze of friends decreases it. We suggest that, in this virtual space, felt presence and subsequent impression management motivations suppress WTP in the felt presence of a group of peers undergoing extraordinary financial distress. As shown in S11 Table, we find no effect of the treatment on how much participants report liking the items, further supporting the notion that differences in payment visibility (rather than in preferences) are driving differences in WTP.

### Heterogeneity by utilization

In this section we consider the role of participants’ heterogenous Venmo use in determining WTP. High-utilization users may be different from other users for two reasons. First, their knowledge of the platform may increase their knowledge of its relevant norms, thereby making them better able to target their impression management efforts. Second, they may have higher WTP when using the platform. Relative to new adopters, high-utilization users have fewer frictions associated with platform use; they therefore have increased convenience and lower pain of payment associated with using Venmo. All else being equal, then, these users’ WTP may be higher.


$$\begin{array}{c} Y_{i,j}=\alpha_{\text{0}}+PaymentFor\overset{\prime }{\underset{i}{m}}\alpha_{1}\;+\;\alpha_{\text{2}}\;Utilization_{i}\;+\;\alpha_{3}PaymentForm_{i}\;\times \;Utilization_{i}\;+\;\alpha_{\text{4}}Item_{j}+\varepsilon_{i,j} \end{array}$$ (2)

Each column in Table 3 reports coefficient estimates from Equation 2, where, as before, *i* represents an individual respondent and *j* represents the lottery item. $Y_{i,j}$ is individual *i*’s WTP for item *j.* As before, item level fixed effects are included in all specifications. Now each column includes a different Venmo utilization variable: in column (1) *Utilization* indicates the amount (in dollars) that the participant reports spending using Venmo in an average month; in column (2) *Utilization* indicates the number of transactions that a participant reports completing in an average month using Venmo; and in column (3) *Utilization* indicates the number of payment methods which the participant reports having linked to their Venmo account.

**Table 3:** Interaction between treatment and Venmo utilization.

	(1)	(2)	(3)
DV: *WTP*	Utilization = *Outflow*	Utilization = *Transactions*	Utilization = *Num. of Linked Payments*
Debit Card	−0.284 (0.273)	−0.281 (0.272)	−0.277 (0.268)
Credit Card	−0.391 (0.282)	−0.364 (0.280)	−0.409 (0.290)
Venmo – Friends	−0.519^*^ (0.270)	−0.608^**^ (0.271)	−0.933^**^ (0.423)
Venmo – Public	−0.128 (0.302)	−0.218 (0.317)	0.166 (0.518)
Utilization	−0.003^**^ (0.001)	−0.013^**^ (0.006)	−0.117 (0.149)
Venmo – Public × Utilization	0.002 (0.003)	0.068^*^ (0.035)	−0.132 (0.234)
Venmo – Friends × Utilization	0.003^*^ (0.002)	0.049^**^ (0.022)	0.380^**^ (0.183)
Item FE	Y	Y	Y
Constant	1.494^***^	1.470^***^	1.587^***^
	(0.230)	(0.230)	(0.368)
Observations	2330	2340	2340
R-Squared	0.043	0.045	0.045

This table reports coefficient estimates from estimating Equation 2. In the above table “Utilization” indicates one of four variables which indicate the intensity of participants’ reported Venmo usage: in column (1) Utilization indicates the amount (in dollars) that the participant reports spending using Venmo on an average month; in column (2) Utilization indicates the number of transactions that a participant reports completing in an average month using Venmo; and in column (3) Utilization indicates the number of payment methods which the participant reports having linked to their Venmo account. Robust standard errors, clustered at the participant level, are reported in parentheses* p < 0.10, ** p < 0.05, *** p < 0.01.

We find that in all specifications, while the statistically significant dampening effect of the Venmo-*Friends* condition on WTP persists, there is now a mitigating effect of participants’ platform utilization. Each additional transaction that an individual has on the platform in the average month diminishes the magnitude of the treatment effect of the Venmo-*Friends* treatment by $0.05 (s.e. = 0.022). The number of platform transactions reported by participants range between zero and 69 (with the average number of transactions being 3.28 and the modal number being two), so for the very highest transacting participants, the net effect of the Venmo-*Friends* treatment is to increase WTP rather than to decrease it. We note, however, that only four participants have enough transactions for this to be the case. Likewise, each additional linked payment (where participants have linked between zero and three payment methods to their Venmo accounts) decreases the treatment effect of the Venmo-*Friends* treatment by $0.38 (s.e. = 0.18). For linked payments, only six participants have linked more than two accounts. The effect of each additional dollar of outflow is quite small but directionally consistent ($0.003; s.e. = 0.002) with the effects of the other utilization variables. We note that for all participants save six, the magnitudes of these utilization effects are small enough that they partially counteract, but do not completely eliminate the treatment effect of the felt presence of friends we describe above. Interestingly, we find that, in the case of the transactions measure, utilization mitigates not just the magnitude of the effect of Venmo-*Friends* treatment, but also the magnitude of the effect of the Venmo-*Public* treatment as well.

Clearly, treatment effects are heterogeneous by platform use. We can only speculate as to why this is the case. One possible explanation is that frequent platform users both know more about, and have increased psychological ownership over, the platform [47]. If this is the case, impression management in this group is more precise and well-informed than that of less frequent users. The mitigation we observe may be a calibration effect as frequent users move their WTP closer to the true norm. Conversely it may also be the case that for more frequent users of a platform, the pain of payment is decreased and convenience is increased, leading to increased WTP. This heterogeneity is a fruitful direction for future research.

### Priming sub-treatment

**Fig 2:**
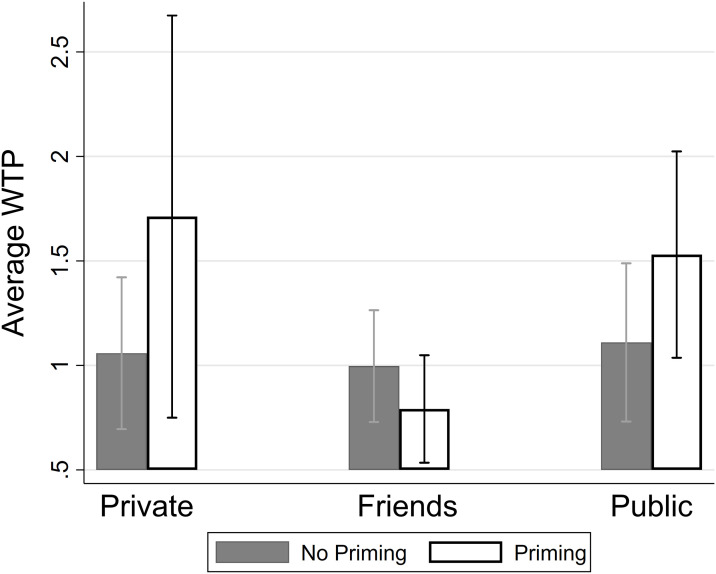
Impact of priming on WTP for each Venmo privacy setting. Mean willingness to pay for each treatment arm (in dollars) with 95% confidence intervals, separately by whether participants view the social feed within Venmo before entering the experiment (“Priming”) or did not view the social feed before the experiment (“No Priming”).

We next consider an exploratory analysis of the effects of reminding participants about the social group to which their transactions will be visible, of making salient their target group by priming respondents by instructing them to view the “social awareness stream” of their Venmo accounts for two minutes before proceeding with the experiment. By viewing this social feed, participants see the usernames, and user-generated descriptions of transactions of everyone who has elected to make their transactions public, as well as of their friends. While these results must be interpreted with caution due to small sample sizes, in the aggregate, we see in Fig 2 that being reminded of others’ consumption increases WTP (by $0.30, s.e. = 0.21). Across the Venmo-*Public* and *Private* treatment groups, we observe a standard effect of priming consumption – viewing the transactions of others increases own WTP (see Fig 2). Participants within the Venmo-*Public* group increase their valuations by, on average, $0.42 (s.e. = 0.32) when primed and participants in the Venmo-*Private* group increase their valuations by an average of $0.65 (s.e. = 0.52) after priming. In the Venmo-*Friends* group, however, this priming intensifies the impression management effect, *lowering* participants’ valuation for goods (by $0.21).

We then estimate the regression shown in Equation 3, separately for each Venmo group (reported separately in the columns of Table 4) where $IsPrimed_{i,v}$ is a dummy variable equal to one if individual *i* in Venmo group *v* was randomly assigned to view their social feed before participating in the valuation exercise. All specifications include item-level fixed effects.

**Table 4:** Impact of priming consumption on WTP to pay across Venmo treatment arms.

DV: *WTP*	(1)	(2)	(3)	(4)	(5)	(6)	(7)	(8)
Priming	0.297	0.158						
	(0.210)	(0.174)						
Venmo-*Private* with Priming			0.654	−0.088				
			(0.524)	(0.428)				
Venmo-*Public* with Priming					0.420	0.251		
					(0.317)	(0.326)		
Venmo-*Friends* with Priming							−0.205	−0.402^**^
							(0.189)	(0.195)
Venmo Usage Controls	N	Y	N	Y	N	Y	N	Y
Item FE	Y	Y	Y	Y	Y	Y	Y	Y
Constant	1.130^***^	1.202^***^	1.051^***^	1.275	1.315^***^	1.612^***^	1.041^***^	0.842^**^
	(0.138)	(0.313)	(0.295)	(0.825)	(0.235)	(0.598)	(0.195)	(0.352)
Observations	1510	1460	490	480	490	470	530	510
R-Squared	0.034	0.041	0.038	0.077	0.108	0.137	0.095	0.171

This table reports the regression coefficients of estimating Equation 3 for all aggregated Venmo treatments (Column 1), and for the separate treatments (Columns 2–4). Robust standard errors, clustered at the participant level, are reported in parentheses^*^ *p* < 0.10, ^**^ *p* < 0.05, ^***^ *p* < 0.01.


$$\begin{array}{c} \overset{k}{\underset{i,v}{Y}}=\alpha_{\text{0}}+\alpha_{\text{1}}IsPrimed_{i,v}+\alpha_{\text{2}}Item_{j}+\alpha_{\text{3}}Utilization_{i}+\varepsilon_{i,v} \end{array}$$ (3)

Column1 reports coefficient estimates associated with estimating Equation 3 across all priming treatments. Columns 3, 5, and 7 report the treatment effect of being primed relative to not being primed for participants in the Venmo-*Private*, Venmo-*Public*, and Venmo-*Friends* groups respectively without any additional controls. As social stream viewing makes further salient the social group to which their transactions will be visible, point estimates suggest that browsing social feeds exacerbates the baseline effect of privacy settings within the platform, regardless of whether those effects cause an increase or decrease in WTP. In Columns 4, 6, and 8 we control for differences in Venmo utilization across groups, which have been found in the previous section to moderate the effect of payment form on WTP.

Consistent with impression management, when controls are included we now see statistically significant evidence that priming increases the magnitude of the impression management effect – when we look within each Venmo treatment group and compare the WTP of participants in this group who have been primed with those who have not, we find suggestive evidence that only in the Venmo-*Friends* treatment group does priming statistically significantly change participants’ WTP. Within the Venmo-*Friends* treatment group, after being primed, participants’ WTP is $0.40 lower (s.e. = 0.20) than among participants who have not been primed. This decrease is relative to other WTP elicitations within the Venmo-*Friends* treatment group which are *already lower* than WTP using other payment forms (by between $0.047 and $0.44 in column 1 of Table 2). This is consistent with a story in which priming has made the audience more salient to participants, leading to amplified impression management effects.

### Heterogeneity across item types

In final exploratory analyses we test whether treatment effects differ across item types, noting that we are highly underpowered to estimate statistically significant effects. We divide the ten items into four categories: 1. *Food/Drinks* (the can of Coca Cola, granola bar, pack of gum, and bottle of coffee), 2. *Office Supplies* (the notebook, pen, and phone wallet), 3. *Toiletries* (the toothpaste and soap), and 4. *COVID-Related* (the cloth face mask). We then re-estimate Equation 1, now omitting item-level fixed effects and including an interaction term between item category and the payment conditions. We anticipate that since digital payments are more commonly used for some categories of items, like food and drinks, there may be a decreased pain of paying when using digital payments for these items.

Estimates in Column 3 of S12 Table show little evidence that the effect of payment form on valuation depends on item type. The only statistically significant interaction variable is for *Venmo Private No Priming* × *COVID*, which means that the mean WTP for *COVID-Related Items* when using the *Venmo Private* setting without priming is $0.88 less than the mean WTP for items in the *Food/Drinks* category (s.e. = 0.28) when using a debit card, approximately a 100% decrease. Wald tests within each item category do not find significant differences in payment form coefficients for each of the five payment groups. Following a regression of payment conditions, product categories (where *Food/Drinks* is the omitted category), and their interactions on WTP, we fail to reject the null hypotheses that the payment form coefficients are equal for *Office Supplies* (*F*(3,233) = 0.47, *p* = 0.70), for *Toiletries* (*F*(3,233) = 1.34, *p* = 0.26) and for *COVID* (*F*(3,233) = 0.22, *p* = 0.88).

### Robustness – randomization inference

Coefficient and standard error estimates of treatment effects estimated using OLS regressions can be sensitive to outliers, a problem which is even more acute in smaller samples like our own. This problem is exacerbated within subsets of samples (e.g., the priming sub-treatment) and in regressions in which an experimental variable is interacted with a non-experimental covariate which contains outliers (which may be the case for the *Utilization* variables) [48]. For our primary results in the sections above, we therefore report the standard OLS *p*-values and the *p*-values from conducting randomization inference tests in Table 5. These tests provide a basis for more accurate inference in the presence of small samples and outlying observations [48].

**Table 5:** Randomization inference *p*-values of main coefficients.

Coefficient	Unadjusted *p*-value	RI adjusted *p*-value
**With Item FEs Only**		
*Venmo – Friends* (estimate shown in Column (1) of Table 2)	0.091	0.046
*Venmo – Friends × Utilization* (where Utilization is defined as Transactions per Month)	0.028	0.014
*Venmo – Friends × Utilization* (where Utilization is defined as Num. of Linked Payments)	0.038	0.085
*Venmo-Friends with Priming* (estimate shown in Column (7) of Table 4)	0.283	0.271
**With Item FEs and Venmo Usage Controls**		
*Venmo – Friends* (estimate shown in Column (2) of Table 2)	0.098	0.053
*Venmo – Friends × Utilization* (where Utilization is defined as Transactions per Month)	0.018	0.259
*Venmo – Friends × Utilization* (where Utilization is defined as Num. of Linked Payments)	0.022	0.099
*Venmo-Friends with Priming* (estimate shown in Column (8) of Table 4)	0.044	0.071

This table reports *p*-values adjusted using the randomization inference procedure outlined in Young (2019). The p-values are calculated from 1,000 random draws and are interpreted in relation to the sharp null hypothesis that there is no treatment effect of the variable listed. Randomization estimates reported here were computed using the package written by Heß (2017).

Randomization inference tests consider the estimated treatment effect under each of many (e.g., 1,000 or more) possible instantiations of random assignment to treatment. For a given null hypothesis, these tests find a distribution of test statistics (in our case regression coefficient estimates) associated with these differing possible assignments to treatment. *P*-values tell us about the relative extremeness of the actual, observed, test statistic in comparison with this distribution.

We find that our main result remains statistically significant when using a randomization inference approach [48,49]. In specifications with only item-level fixed effects or in specifications including both item-level fixed effects and Venmo usage controls, we find evidence for robustness of the claim that being assigned to the Venmo-*Friends* treatment lowers participant WTP. We also see that the treatment effect heterogeneity induced by participants’ utilization patterns persists in the case of considering the number of linked payments as the proxy for utilization, but the transactions per month utilization estimate is less robust. This is likely driven by the fact that this latter measure includes outliers and is much more varied than is the measure of the number of linked payments. The statistical significance of the estimates of the effect of the priming sub-treatment remains largely unchanged.

## Discussion and implications

In a real-stakes experiment conducted during Covid-19 we randomly assign 261 college students to one of five payment forms: either credit card, debit card, or one of three social settings within *Venmo*. By doing so, we construct three possible audiences for participants’ transactions: 1) that of the participant and the experimenter (induced by the credit, debit, and Venmo-*Private* conditions); 2) that of the participant and a subset of their peers (induced by the Venmo-*Friends* condition) and 3) that of the participant and all other public Venmo users (induced by the Venmo-*Public* condition). We then elicit from these students their willingness to pay for each of ten everyday household items using a BDM mechanism. This research considers WTP using mobile payments in the felt presence of others. We extend work considering the role of social influences in retail environments, which has been predominantly conducted in the physical presence of others. We also contribute to the literature examining differences in WTP across payment forms.

We find that the audience to which participant payments will be visible matters; participants are willing to pay statistically significantly less ($0.44, *p* = 0.091), on average, for items when these purchases will be visible to friends than when purchases are private. Participants’ increased use of the platform partially mitigates these effects. While this result must be interpreted with caution due to small sample sizes, we also find that when priming has made the relevant peer group more salient to participants, the effect of consuming in front of peers is amplified. These findings highlight the importance of considering the felt presence of others, in addition to more well-documented features such as convenience, transparency and pain of payment, when understanding the differences in WTP across payment forms.

Our experiment was conducted in the United States during Covid-19, a time of unprecedented economic disruption where consumers adjusted not only their WTP, but also their consumption bundles and the manner in which they paid for this consumption. During this time, consumers initially adjusted their purchases by spending more on retail and food items [27]. By the time of our study, however, consumers had sharply decreased their overall spending [27]. During Covid, consumers were found to have increased WTP for protective items such as disposable face masks [50], food [51], as well as for a variety of household items such as card games, soft drinks, and napkins [52]. Consistent with our findings, it has also been found that consumers displayed higher WTP when using contactless payment methods [53]. In the phase of Covid in which this experiment was conducted, global consumption patterns were characterized by atypical levels of stockpiling [54], panic-driven purchases [55], and impulsive purchases [56–57]. Here, we have considered the unplanned, impulse purchases which were possible as part of our experiment. Such impulse purchases been shown elsewhere to have been affected by peer behavior during Covid [56].

We have considered the role of payment forms, and their implied audience, on WTP during a time in which the topography of payment form utilization in the United States was rapidly changing. Unlike previous disruptive events facing consumers in the United States such as other pandemics and epidemics (e.g., HIV/AIDS and H1N1), natural disasters (e.g., hurricane Katrina), and economic crises (e.g., the Great Recession), the Covid-19 pandemic was global in nature, and occurred during a time of significant technological advancement (a period which has been called a “digital transformation”) [58]. Between 2019 and 2020 (the first year of the pandemic), the share of payments made using cash in the U.S. dropped sharply from 26% to 19% [59], only slightly increasing in 2021 (to 20%) and continuing to remain below pre-pandemic levels. Consumers’ holdings of cash, unlike their usage of it, remained elevated throughout the pandemic, suggesting that consumers saw cash increasingly as a store of value in an uncertain economic time, and less as a medium with which to conduct transactions [59]. At the same time, lockdowns and a drive toward contactless payments accelerated the transition to mobile payments. Between October 2019 and October 2020, the share of consumers who had made at least one mobile payment in the preceding year increased from 37.5 percent to 46.1 percent [60]. The most commonly used among these mobile payments platforms were PayPal (utilization of which increased to 42% of consumers in 2020 from 38% of consumers in 2019), Venmo (utilization of which increased to 24% of consumers in 2020 from 15% of consumers in 2019), and Zelle (which increased to 17% of consumers in 2020 from 11% of consumers in 2019). Among these mobile payment platforms, Venmo’s *social awareness stream* uniquely presented consumers with a differentiated social environment.

We contribute to work which, across a variety of domains, has found that the social dimensions of consumers’ choice environment mattered for behavior during Covid. For example, Rabb et al. conduct a survey which, like our experiment, highlights the special role of close contacts during Covid. In their work exploring the role of social norms on vaccination intentions, the authors find that the perceived vaccination norm among the peers closest to the participant, e.g., friends and family, strongly predicted the participant’s own intention to vaccinate (even after controlling for things such as disease risk and trust in science). As the social group expanded to the participant’s neighborhood, city and state, the perceived norm among that group became less predictive of the participant’s own intentions [61]. Other work includes a study of Chinese consumers, among which it was found that peer pressure increased consumers’ WTP for vaccines [62]. In Austria, social norms increased compliance with preventive behaviors such as maintaining social distance, wearing face masks or minimizing social distance [63]. In fact, social norms have not only been found to be associated with preventive behaviors but to causally impact them in an experimental setting [64]. Social visibility has also been found to matter during Covid for purchases unrelated to health and disease prevention. In a study of panic-driven buying in Singapore which used a structural equation modelling framework, the authors considered multiple different behavioral constructs and found that the strongest predictor of panic-buying was normative social influence [65]. An experiment conducted in China found that the perception of social isolation lead to a higher intention to purchase conspicuously-branded luxury goods. [13]

Within the work which considers the importance of social visibility during Covid, our paper specifically contributes to work which finds that impression management behaviors were key behavioral explanators during this time. In an important test of the role of impression management in reporting during Covid-19, Daoust et al [66] run a “face-saving” experiment in 12 countries during Covid, in which they compare stated compliance with protective behaviors, randomly varying the social sanction which noncompliance represents. They find that once noncompliance has been normalized, thereby relaxing impression management concerns, participants report statistically significantly less compliance with Covid-19 preventive measures. In other studies conducted during Covid, impression management concerns have been shown to either predict or moderate: compliance with guidelines [67], intentions to wear a mask [68,69], sharing of public health information via social media [70], workplace preventative behavior [71], worker fatigue during virtual meetings [72], and the authenticity of images posted to Instagram accounts [73].

We interpret our results as suggesting that in this setting, impression management concerns motivate college students to *lower* their spending. We speculate that due to the period of contemporaneous economic hardship, frugality was the operating norm among students at that time. While the effects we find are consistent with other work studying impression management, we cannot provide direct measures of individuals’ desired impression, the importance to them of the friends to which their transactions would be visible, or of the operative norm within this friend group. There remain, therefore, alternate mechanisms which we cannot rule out.

The first possible mechanism is that impression management works in this setting, not through conformance with some norm, but through individuals’ agency-communion orientation. Kurt et al. find decreased consumption behavior for communal consumers (proxied for by female gender) when a friend is present [15]. Future work could measure participants’ agency-communion orientation and explore the relationship between this measure and WTP.

The next possible mechanism is the embarrassment of making an unusual purchase in the felt presence of others [74]. In an environment where most transactions occur between peers and are for food or transportation, it is possible that participants felt embarrassed to pay for items within the context of our experiment (by sending money to our account “VenmoWTPResearch”). It is plausible then that consumers might be more embarrassed to reveal their experimental participation to their friends than to the public at-large. Consistent with our usage heterogeneity findings, Dahl, Manchanda and Argo find that as consumers’ familiarity with a purchase increases, their embarrassment with making this purchase decreases [48]. We cannot rule out that embarrassment might be working in place of, or in concert with, impression management in our findings.

Future work can disambiguate mechanisms by collecting behavioral measures directly related to impression management and pain of payment within the context of a similar experiment. Fruitful extensions could also explore WTP-relevant aspects of the decision environment we did not experimentally vary. There are at least three important dimensions which should be explored. First, the nature of the good. The purchasing decisions within our study are unplanned impulse purchases; the interaction between social groups and mobile payments might differ when purchasing is being done deliberatively. We considered household items, but perhaps the treatment effects would differ if goods were more conspicuous in nature. Second, we note that the user account that individuals paid for the items within the Venmo treatments may have interacted with participant norms differently than would have a more naturalistically named Venmo recipient account. Finally, there is also an important communicative aspect of digital payment platforms, particularly of Venmo. Users report putting a lot of thought into captions of their transactions, and use the platform for its social benefits as well as for its purely task-driven use [43]. Subsequent studies might disentangle purely consumption-driven behavior from instrumental or communicative behaviors.

## Supporting information

S1 Fig Experimental screenshots. (PDF)

S2 Table Estimates from regression of WTP on payment form including the full ITT sample. This table reports coefficient estimates from estimating equation one where the dependent variable of interest is an individual’s WTP including the full intent-to-treat sample (rather than the cleaned sample). Estimates are reported with and without demographic and Venmo usage controls. All specifications include item-level fixed effects. Venmo-Private is the omitted category in all specifications. Robust standard errors, clustered at the participant level, are reported in parentheses. *** p < 0.01, ** p < 0.05, * p < 0.1.
(DOCX)

S3 Table Interaction between treatment and Venmo utilization including the full ITT sample. This table reports coefficient estimates from estimating Equation 2 for the full intent-to-treat sample (rather than the cleaned sample). In the above table “Utilization” indicates one of four variables which indicate the intensity of participants’ reported Venmo usage: in column (1) Utilization indicates the amount (in dollars) that the participant reports spending using Venmo on an average month; in column (2) Utilization indicates the number of transactions that a participant reports completing in an average month using Venmo; and in column (3) Utilization indicates the number of payment methods which the participant reports having linked to their Venmo account. Robust standard errors, clustered at the participant level, are reported in parentheses* p < 0.10, ** p < 0.05, *** p < 0.01.
(DOCX)

S4 Table Impact of priming consumption on WTP to pay across Venmo treatment arms including the full ITT sample. This table reports the regression coefficients of estimating Equation 3 for all aggregated Venmo treatments (Column 1), and for the separate treatments (Columns 2–4) including the full intent-to-treat sample (rather than the cleaned sample). Robust standard errors, clustered at the participant level, are reported in parentheses^*^ *p* < 0.10, ^**^ *p* < 0.05, ^***^ *p* < 0.01.
(DOCX)

S5 Table Pairwise tests of equality of means across treatment groups. This table reports pairwise t-tests of differences in mean participant characteristics in the cleaned sample. Pairwise differences which are statistically significantly different from zero are indicated by stars. “C” indicates the *Credit* treatment group, “D” represents the Debit treatment group, “VFriends” indicates the Venmo-Friends treatment group, “VPublic” indicates the *Venmo-Public* treatment group, “VPrivate” indicates the *Venmo-Private* treatment group.
(TIF)

S6 Table Estimation of Equation 1 for COVID items only. (DOCX)

S7 Table Estimation of Equation 1 for office items only. (DOCX)

S8 Table Estimation of Equation 1 for toiletry items only. (DOCX)

S9 Table Estimation of Equation 1 for food items only. (DOCX)

S10 Fig Mean WTP and participant rating for each treatment arm (in dollars) with 95% confidence intervals. This figure illustrates mean willingness to pay (dark gray), and mean preference ratings (light gray) across all items for each treatment group. 95% confidence intervals are shown with standard errors clustered at the participant level.
(TIF)

S11 Table Estimation of The Effect of Payment Form on Participant Ratings. (DOCX)

S12 Table Regression of WTP on payment form, item category, and their interaction. This table includes regression coefficients of estimations of WTP on payment form, item category, and their interaction. Robust standard errors, clustered at the participant level, are in brackets. *** p < 0.01, ** p < 0.05, * p < 0.1.
(DOCX)
