# Supplementary material for: Paying in public: Peer effects, impression management, and willingness to pay on digital payment platforms
Source: PLoS One. 2026 Jul 1;21(7):e0340550. doi: 10.1371/journal.pone.0340550 (PMC13322516; doi:10.1371/journal.pone.0340550)
Supplement: S1 Fig — (PDF) [file pone.0340550.s001.pdf]

This file includes screenshots for the experiment reported in Kiernan & Debnam Guzman (2026)

Details about Payment Processing

Those assigned to Venmo are asked to pay a Venmo account created for the experiment (@WillingnessToPayResearch). Those assigned to pay with debit or credit are asked to pay through Square, a secure 3rd party credit card processing service, which opens in a new tab from the online experiment. If participants do not feel comfortable, they are allowed to abstain from paying; only 1.6% of participants who won at least one lottery chose not to pay. If a participant does not pay, they are asked why; the provided options are 1) feel uncomfortable providing payment information online, 2) do not think the item was worth the randomly drawn price, 3) never planned to submit a payment, or 4) do not possess the assigned payment form. The only response that does not compromise the participant's WTP estimates is the first option, that they did not feel comfortable entering their payment information online. The other answers suggest that the participant either misreported personal information or inaccurately estimated their WTP, both of which question the integrity of the data they provided. Thus, if a participant selects anything other than feeling uncomfortable submitting a payment online, the associated observation is dropped from the dataset.

Additional Details about Data Cleaning

To include only incentivized reports of willingness to pay, we drop further observations from participants who misreported that they paid for their winning item (n = 6) or who did not pay for any reason other than security concerns (n = 1). We also drop observations from participants who responded "$0" for all of their WTP estimates (n = 11), observations of any participants whose answers to the initial screening questions were inconsistent with their answers to final demographic questions (n = 6), and we drop both observations from one participant who completed the survey twice (n = 2).

Screenshot 1: An informed consent form is the first page of the experiment. This is followed by screening questions about age, college attendance, Venmo usage, and debit/credit card possession.

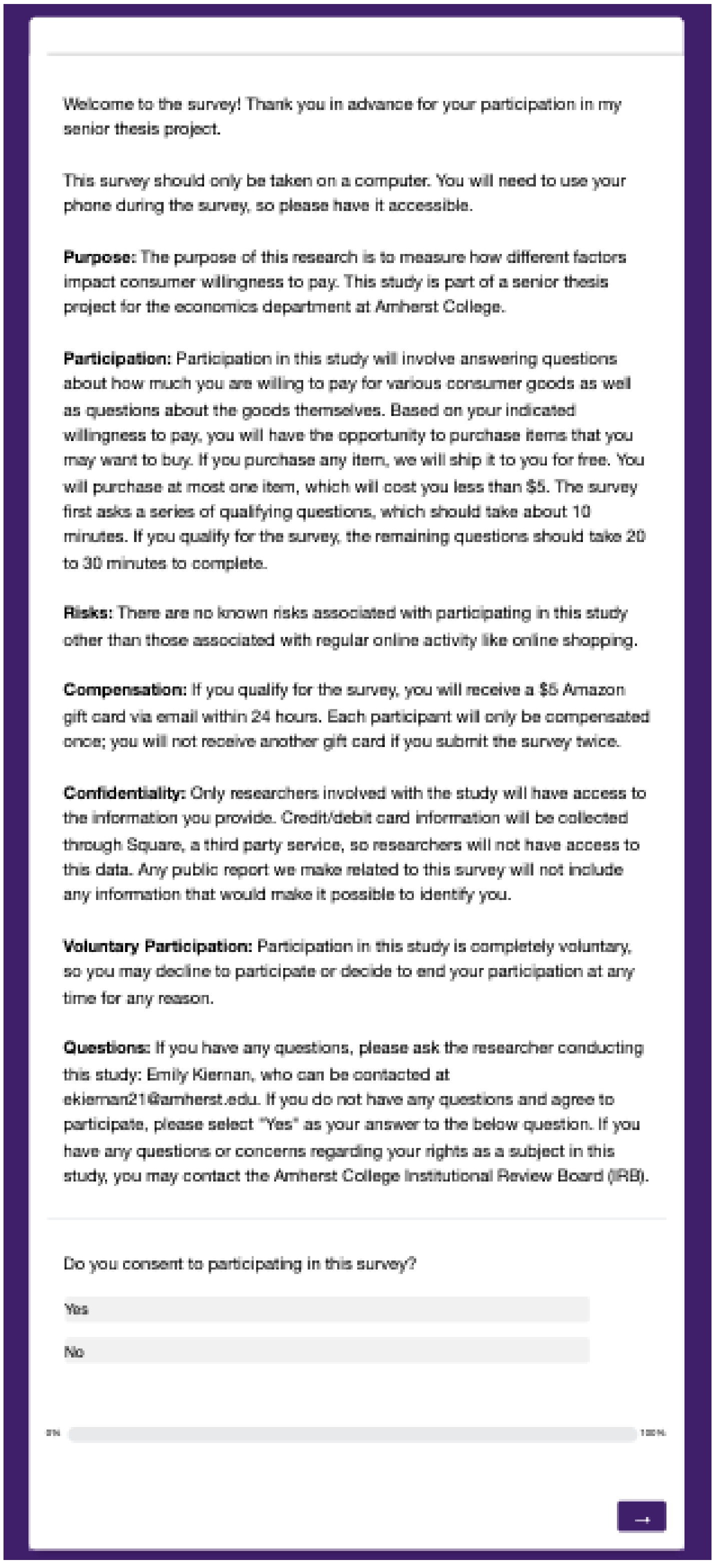

Welcome to the survey! Thank you in advance for your participation in my senior thesis project.

This survey should only be taken on a computer. You will need to use your phone during the survey, so please have it accessible.

Purpose: The purpose of this research is to measure how different factors impact consumer willingness to pay. This study is part of a senior thesis project for the economics department at Amherst College.

Participation: Participation in this study will involve answering questions about how much you are willing to pay for various consumer goods as well as questions about the goods themselves. Based on your indicated willingness to pay, you will have the opportunity to purchase items that you may want to buy. If you purchase any item, we will ship it to you for free. You will purchase at most one item, which will cost you less than $5. The survey first asks a series of qualifying questions, which should take about 10 minutes. If you qualify for the survey, the remaining questions should take 20 to 30 minutes to complete.

Risks: There are no known risks associated with participating in this study other than those associated with regular online activity like online shopping.

Compensation: If you qualify for the survey, you will receive a $5 Amazon gift card via email within 24 hours. Each participant will only be compensated once; you will not receive another gift card if you submit the survey twice.

Confidentiality: Only researchers involved with the study will have access to the information you provide. Credit/debit card information will be collected through Square, a third party service, so researchers will not have access to this data. Any public report we make related to this survey will not include any information that would make it possible to identify you.

Voluntary Participation: Participation in this study is completely voluntary, so you may decline to participate or decide to end your participation at any time for any reason.

Questions: If you have any questions, please ask the researcher conducting this study: Emily Kiernan, who can be contacted at ekiernan21@amherst.edu. If you do not have any questions and agree to participate, please select "Yes" as your answer to the below question. If you have any questions or concerns regarding your rights as a subject in this study, you may contact the Amherst College Institutional Review Board (IRB).

Do you consent to participating in this survey?

Yes

No

0% 100%

→

Screenshot 2: After the screening questions, participants are assigned to one of three payment forms: debit, credit, or Venmo.

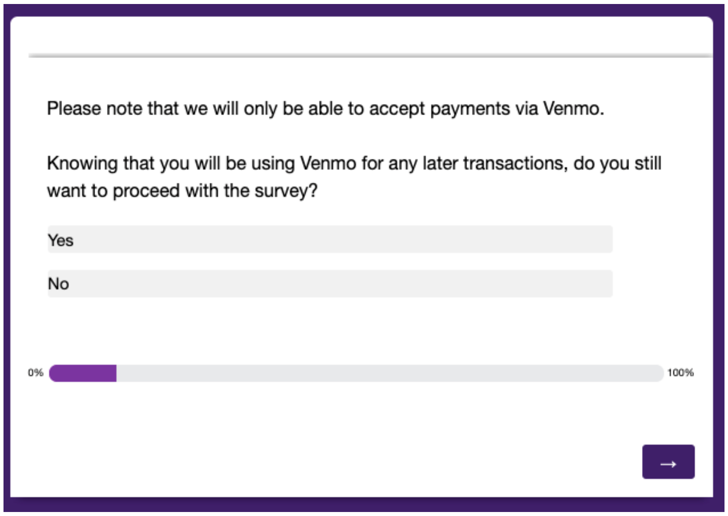

Please note that we will only be able to accept payments via Venmo.

Knowing that you will be using Venmo for any later transactions, do you still want to proceed with the survey?

Yes

No

0% 100%

→

Screenshot 3: Those assigned to Venmo are then asked to change their default privacy setting to their randomly assigned setting of either *Private*, *Friends Only*, or *Public*.

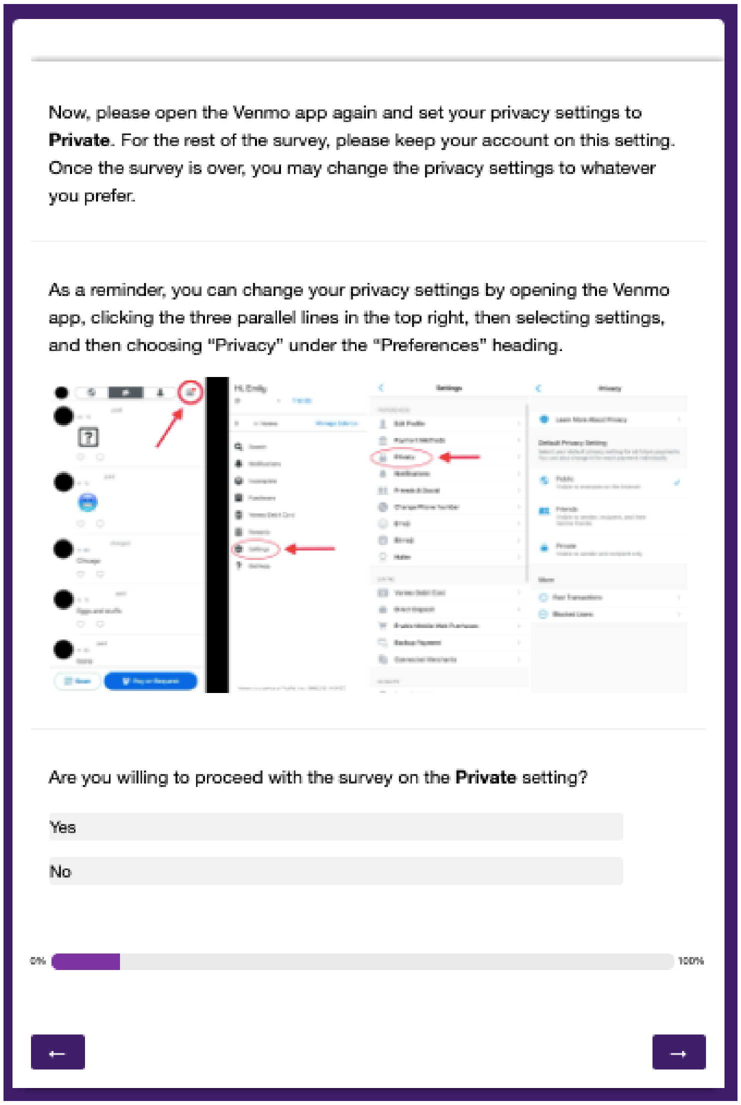

Now, please open the Venmo app again and set your privacy settings to **Private**. For the rest of the survey, please keep your account on this setting. Once the survey is over, you may change the privacy settings to whatever you prefer.

As a reminder, you can change your privacy settings by opening the Venmo app, clicking the three parallel lines in the top right, then selecting settings, and then choosing "Privacy" under the "Preferences" heading.

Are you willing to proceed with the survey on the **Private** setting?

Yes

No

0% 100%

← →

Screenshot 4: Participants assigned to Venmo are then randomly assigned to either priming or no priming. Those assigned to priming are asked to scroll through the *Public* Venmo feed for two minutes while those not assigned to priming simply proceed with the survey.

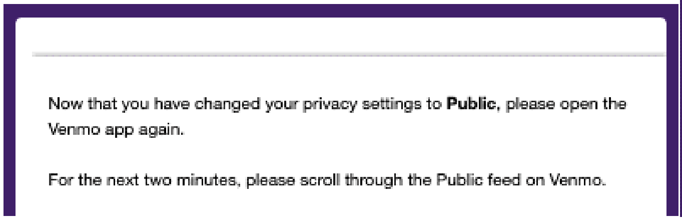

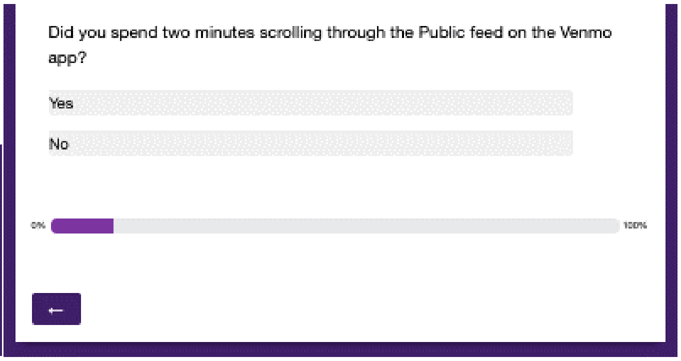

Screenshot 5: Next, the BDM lotteries are explained. A subsequent attention check question ensures participants understand how the lotteries work: “If you win the lottery, what is the price you will have to pay?” (Correct answer: “The randomly drawn purchase price, which is less than or equal to your stated maximum price.”)

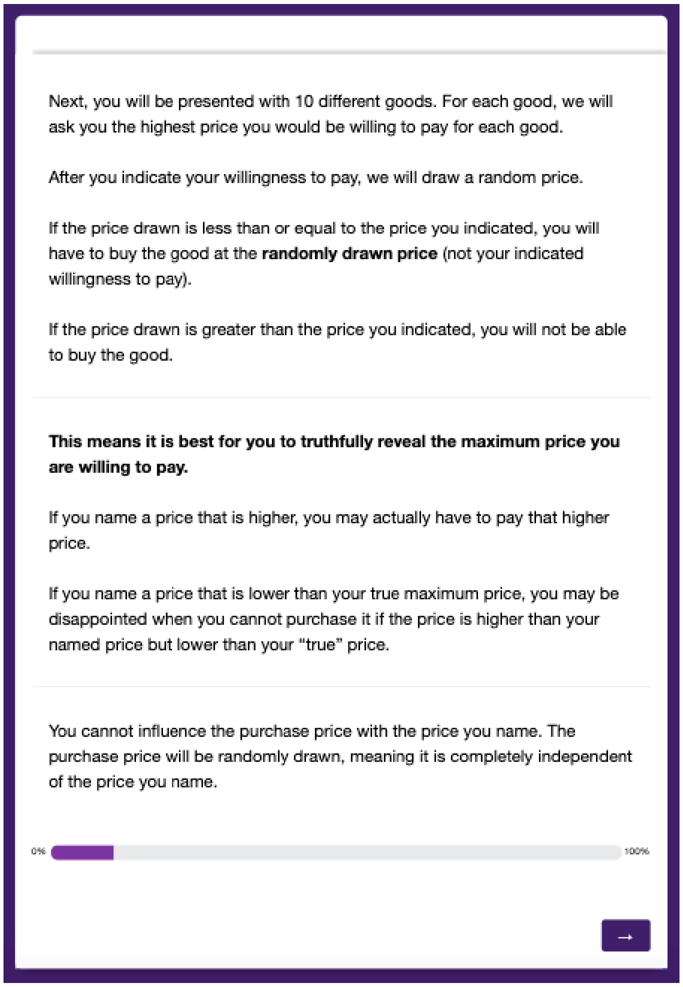

Screenshot 6: After participants confirm that they understand the BDM lottery process, they are told a maximum of one item will be purchased. This is followed by an attention check question: “What is the maximum number of goods you will be required to purchase?” (Correct answer: 1)

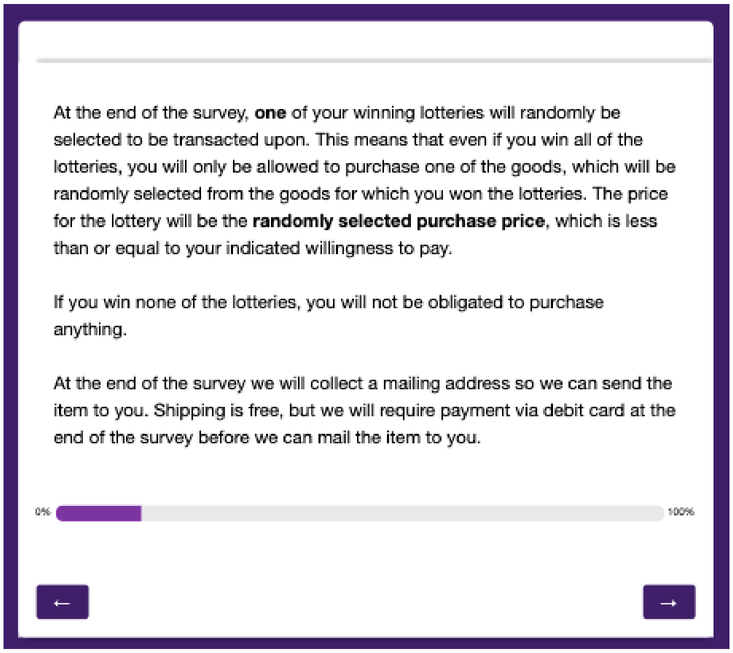

Screenshot 7: Once participants understand how the lotteries work, they are asked to name their maximum price for each of the ten items. This example shows the WTP elicitation for the pack of gum. The order of the ten items is randomized for each participant.

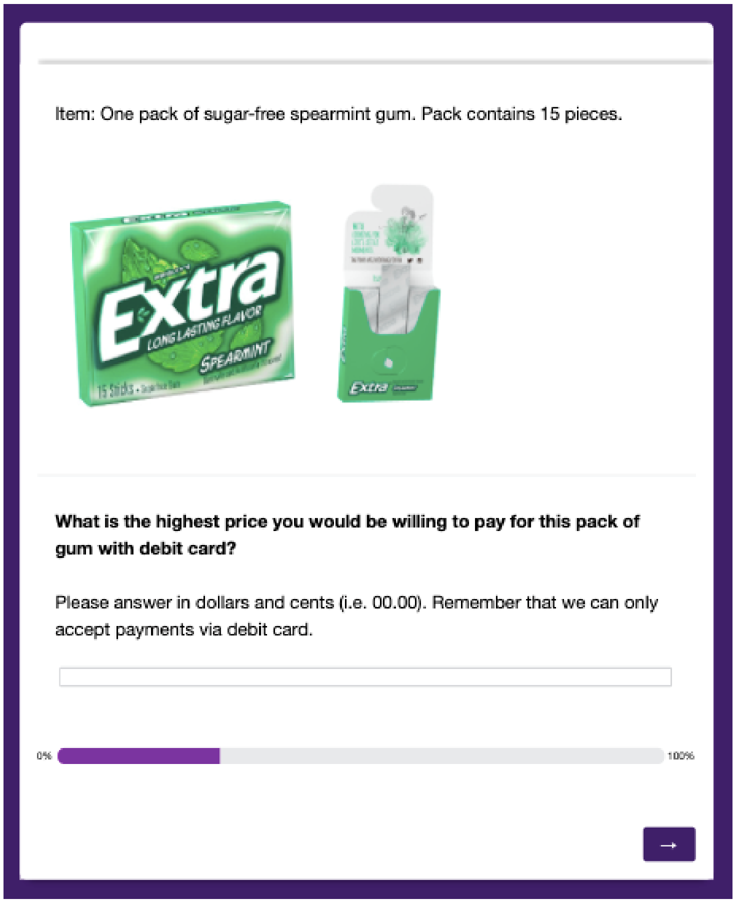

Screenshot 8: After the initial WTP estimate for each of the ten items, participants are given the option to revise their indicated maximum price.

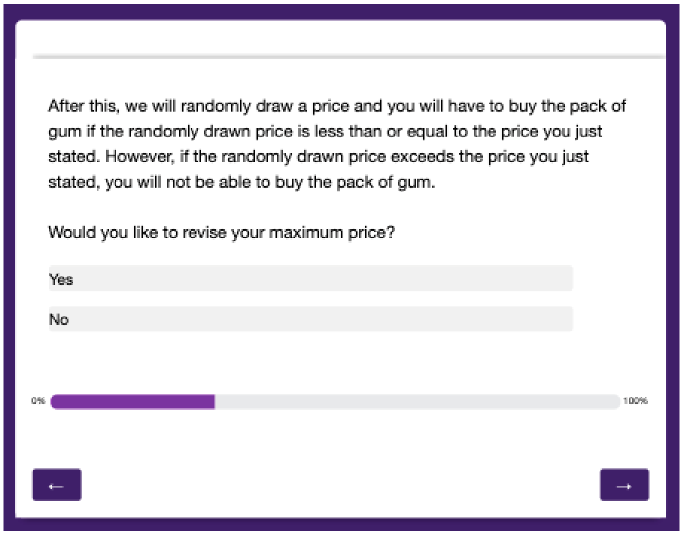
After this, we will randomly draw a price and you will have to buy the pack of gum if the randomly drawn price is less than or equal to the price you just stated. However, if the randomly drawn price exceeds the price you just stated, you will not be able to buy the pack of gum.

Would you like to revise your maximum price?

Yes

No

0% 100%

← →

Screenshot 9: After participants indicate their WTP for a given item (regardless of whether they chose to revise), we ask follow-up questions regarding their preferences about the item.

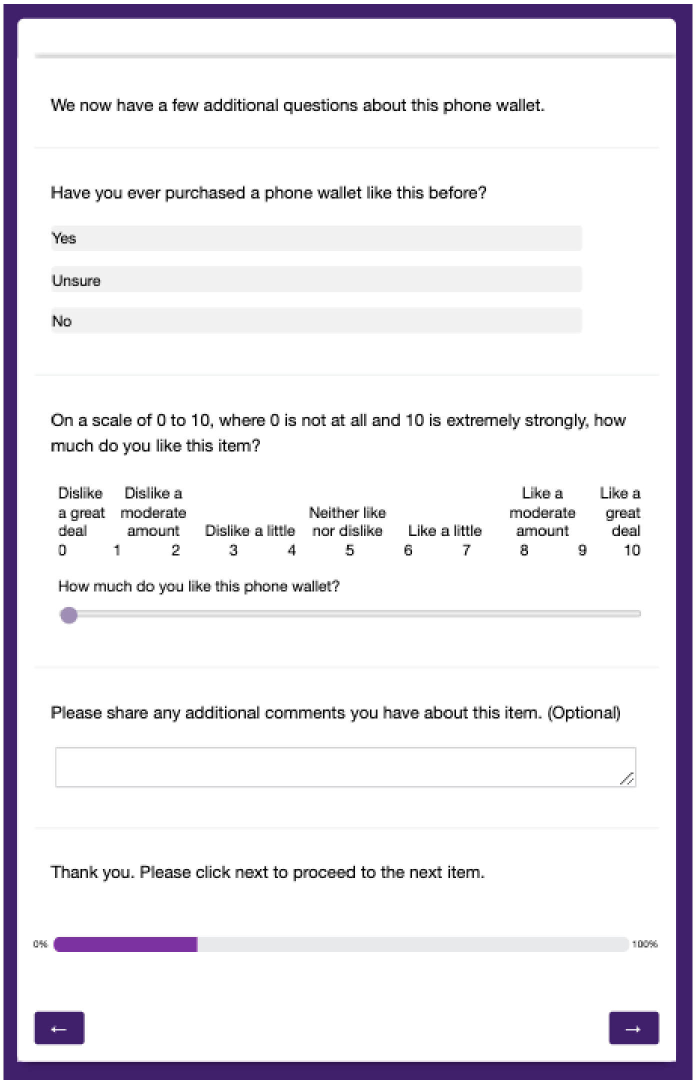
We now have a few additional questions about this phone wallet.

Have you ever purchased a phone wallet like this before?

Yes

Unsure

No

On a scale of 0 to 10, where 0 is not at all and 10 is extremely strongly, how much do you like this item?

Dislike a great deal		Dislike a moderate amount		Dislike a little		Neither like nor dislike		Like a little		Like a moderate amount		Like a great deal
0	1	2	3	4		5	6	7	8	9	10

How much do you like this phone wallet?

Please share any additional comments you have about this item. (Optional)

Thank you. Please click next to proceed to the next item.

0% 100%

← →

Screenshot 10: After completing the WTP estimates and follow-up questions for all ten items, a random price between $1 and $5 is selected for each item.

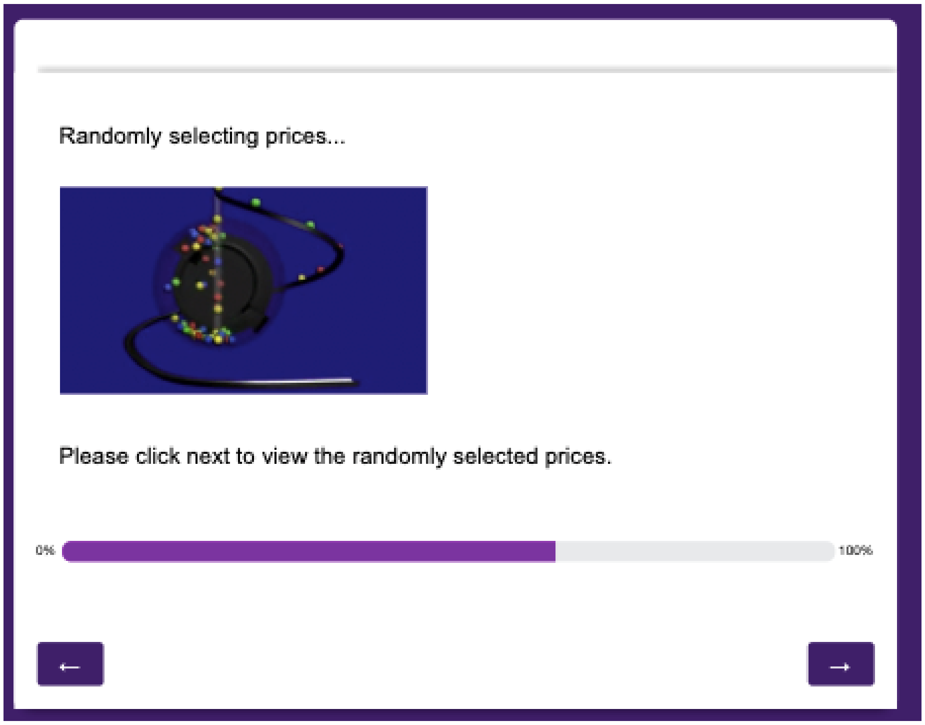

Screenshot 11: Participants are shown a summary of the ten random prices.

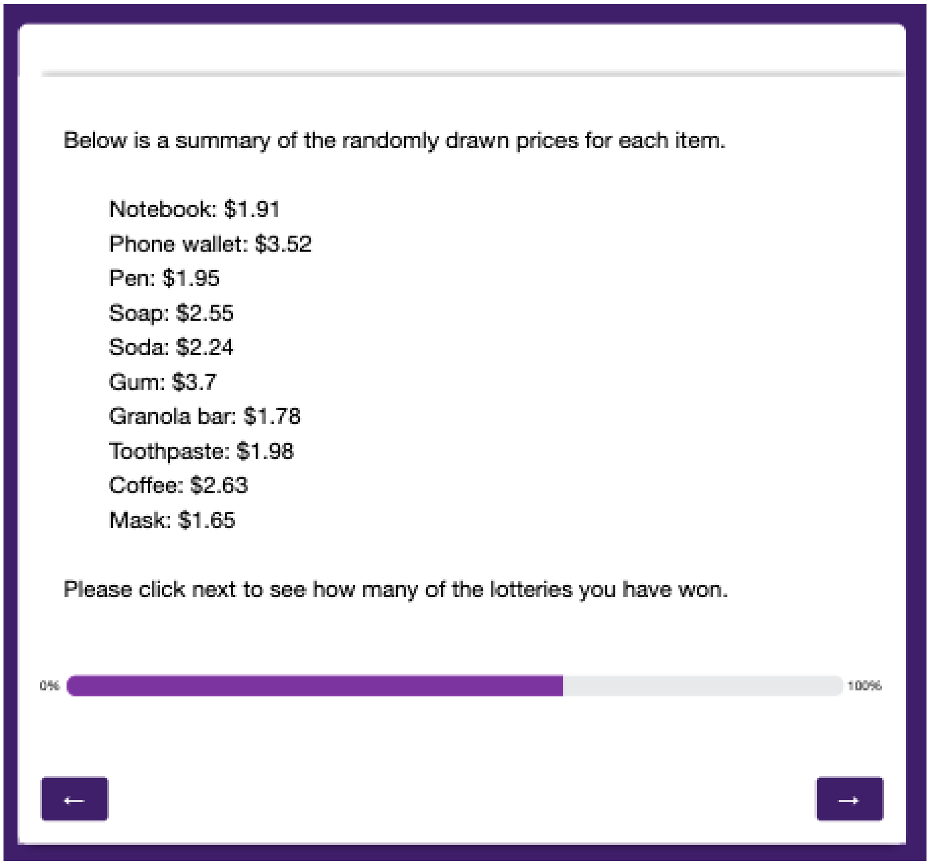

Screenshot 12: After the random selection of prices, participants are shown a table that summarizes the random prices, their indicated WTP for each item, and whether they won each lottery. Participants who won one lottery are asked to purchase that item and participants who won multiple lotteries are asked to purchase one item randomly selected from all their winning items. Participants who won zero lotteries just proceed to the final demographic questions.

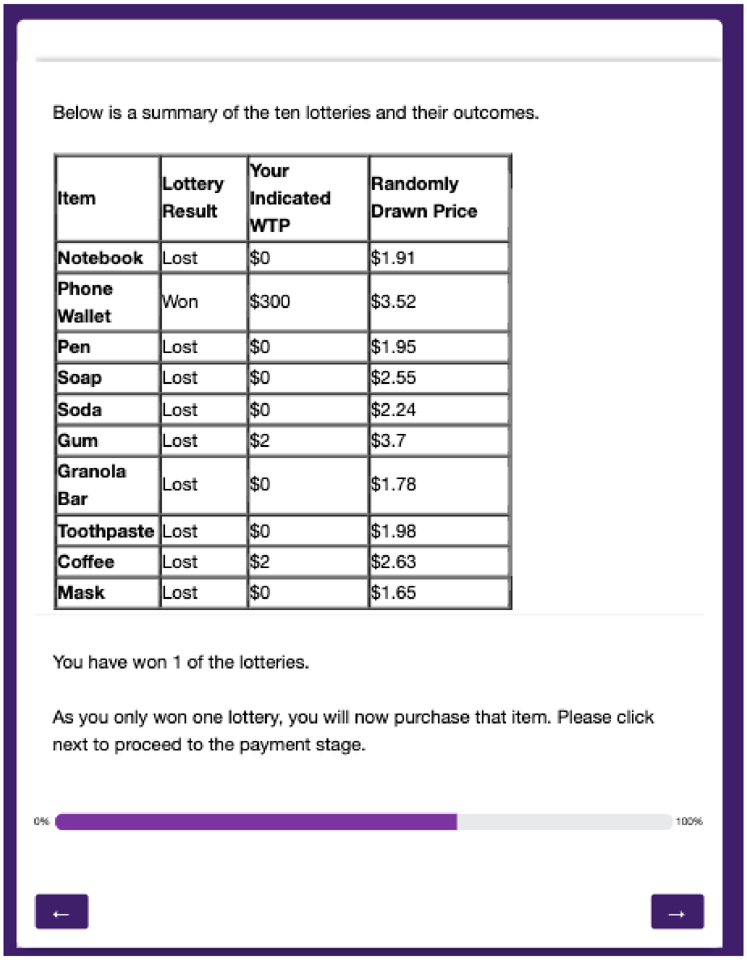
Below is a summary of the ten lotteries and their outcomes.

Item	Lottery Result	Your Indicated WTP	Randomly Drawn Price
**Notebook**	Lost	$0	$1.91
**Phone Wallet**	Won	$300	$3.52
**Pen**	Lost	$0	$1.95
**Soap**	Lost	$0	$2.55
**Soda**	Lost	$0	$2.24
**Gum**	Lost	$2	$3.7
**Granola Bar**	Lost	$0	$1.78
**Toothpaste**	Lost	$0	$1.98
**Coffee**	Lost	$2	$2.63
**Mask**	Lost	$0	$1.65

You have won 1 of the lotteries.

As you only won one lottery, you will now purchase that item. Please click next to proceed to the payment stage.

0% 100%

← →

Screenshot 13: The final page of the survey debriefs participants. All participants, regardless of whether they purchased an item, receive a $5 Amazon e-gift card within 24 hours of completing the experiment.

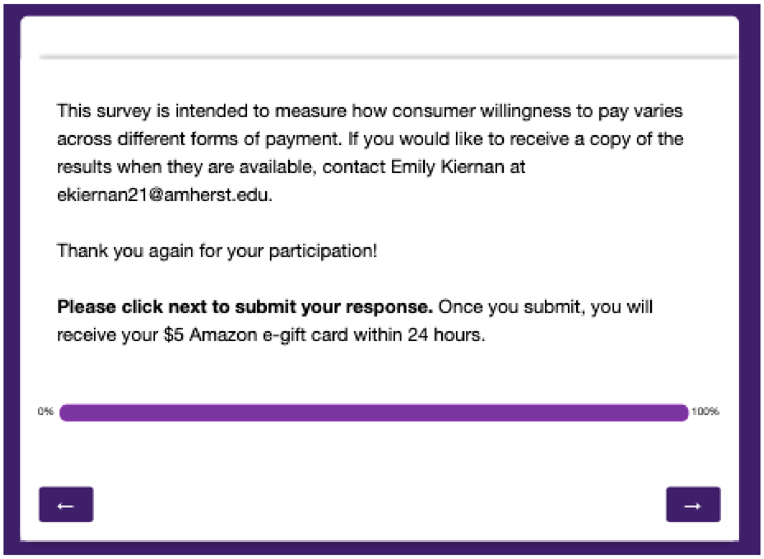
This survey is intended to measure how consumer willingness to pay varies across different forms of payment. If you would like to receive a copy of the results when they are available, contact Emily Kiernan at ekiernan21@amherst.edu.

Thank you again for your participation!

Please click next to submit your response. Once you submit, you will receive your $5 Amazon e-gift card within 24 hours.

0% 100%

← →
