# Supplementary material for: Paying in public: Peer effects, impression management, and willingness to pay on digital payment platforms
Source: PLoS One. 2026 Jul 1;21(7):e0340550. doi: 10.1371/journal.pone.0340550 (PMC13322516; doi:10.1371/journal.pone.0340550)
Supplement: S2 Table — This table reports coefficient estimates from estimating equation one where the dependent variable of interest is an individual’s WTP including the full intent-to-treat sample (rather than the cleaned sample). Estimates are reported with and without demographic and Venmo usage controls. All specifications include item-level fixed effects. Venmo-Private is the omitted category in all specifications. Robust standard errors, clustered at the participant level, are reported in parentheses. *** p < 0.01, ** p < 0.05, * p < 0.1. (DOCX) [file pone.0340550.s002.docx]

| DV: *WTP* | (1) | (2) | (3) | (4) |
| --- | --- | --- | --- | --- |
| Debit Card | -0.287 | -0.240 | -0.380 | -0.334 |
|  | (0.255) | (0.234) | (0.283) | (0.269) |
|  |  |  |  |  |
| Credit Card | -0.364 | -0.275 | -0.361 | -0.262 |
|  | (0.267) | (0.244) | (0.288) | (0.266) |
|  |  |  |  |  |
| Venmo - Friends | -0.425^*^ | -0.401^*^ | -0.532^**^ | -0.476^*^ |
|  | (0.243) | (0.240) | (0.265) | (0.262) |
|  |  |  |  |  |
| Venmo - Public | 0.003 | -0.036 | -0.018 | -0.066 |
|  | (0.275) | (0.254) | (0.303) | (0.296) |
| Demographic Controls | N | N | Y | Y |
| Venmo Usage Controls | N | Y | N | Y |
| Item FE | Y | Y | Y | Y |
| Constant | 1.374^***^ | 1.398^***^ | 2.040 | 1.742 |
|  | (0.209) | (0.285) | (1.291) | (1.340) |
| Observations | 2520 | 2450 | 2200 | 2140 |
| R-Squared | 0.041 | 0.045 | 0.045 | 0.048 |

Standard errors in parentheses

^*^ *p* < 0.10, ^**^ *p* < 0.05, ^***^ *p* < 0.01
