# Supplementary material for: Paying in public: Peer effects, impression management, and willingness to pay on digital payment platforms
Source: PLoS One. 2026 Jul 1;21(7):e0340550. doi: 10.1371/journal.pone.0340550 (PMC13322516; doi:10.1371/journal.pone.0340550)
Supplement: S3 Table — This table reports coefficient estimates from estimating Equation 2 for the full intent-to-treat sample (rather than the cleaned sample). In the above table “Utilization” indicates one of four variables which indicate the intensity of participants’ reported Venmo usage: in column (1) Utilization indicates the amount (in dollars) that the participant reports spending using Venmo on an average month; in column (2) Utilization indicates the number of transactions that a participant reports completing in an average month using Venmo; and in column (3) Utilization indicates the number of payment methods which the participant reports having linked to their Venmo account. Robust standard errors, clustered at the participant level, are reported in parentheses* p < 0.10, ** p < 0.05, *** p < 0.01. (DOCX) [file pone.0340550.s003.docx]

| DV: *WTP* | (1) | (2) | (3) |
| --- | --- | --- | --- |
|  |  |  |  |
| Debit Card | -0.288 | -0.286 | -0.287 |
|  | (0.255) | (0.255) | (0.255) |
|  |  |  |  |
| Credit Card | -0.330 | -0.304 | -0.383 |
|  | (0.266) | (0.265) | (0.278) |
|  |  |  |  |
| Venmo - Friends | -0.510^**^ | -0.590^**^ | -0.953^**^ |
|  | (0.252) | (0.254) | (0.416) |
|  |  |  |  |
| Venmo - Public | -0.116 | -0.096 | 0.125 |
|  | (0.286) | (0.309) | (0.505) |
|  |  |  |  |
| Utilization | -0.003^**^ | -0.013^**^ | -0.124 |
|  | (0.001) | (0.006) | (0.154) |
| Venmo - Public X Utilization | 0.002  (0.003) | 0.033  (0.041) | -0.094  (0.233) |
| Venmo - Friends X Utilization | 0.004^**^  (0.002) | 0.048^**^  (0.021) | 0.411^**^  (0.186) |
|  |  |  |  |
| Item FE | Y | Y | Y |
| Constant | 1.435^***^ | 1.408^***^ | 1.540^***^ |
|  | (0.215) | (0.215) | (0.363) |
| Observations | 2500 | 2510 | 2520 |
| R-Squared | 0.041 | 0.043 | 0.044 |

Standard errors in parentheses

^*^ *p* < 0.10, ^**^ *p* < 0.05, ^***^ *p* < 0.01
