# Supplementary material for: Paying in public: Peer effects, impression management, and willingness to pay on digital payment platforms
Source: PLoS One. 2026 Jul 1;21(7):e0340550. doi: 10.1371/journal.pone.0340550 (PMC13322516; doi:10.1371/journal.pone.0340550)
Supplement: S4 Table — This table reports the regression coefficients of estimating Equation 3 for all aggregated Venmo treatments (Column 1), and for the separate treatments (Columns 2–4) including the full intent-to-treat sample (rather than the cleaned sample). Robust standard errors, clustered at the participant level, are reported in parentheses* p < 0.10, ** p < 0.05, *** p < 0.01. (DOCX) [file pone.0340550.s004.docx]

|  | (1) | (2) | (3) | (4) | (5) | (6) | (7) | (8) |
| --- | --- | --- | --- | --- | --- | --- | --- | --- |
|  | WTP | WTP | WTP | WTP | WTP | WTP | WTP | WTP |
| Priming | 0.233 | 0.129 |  |  |  |  |  |  |
|  | (0.201) | (0.170) |  |  |  |  |  |  |
| Venmo-*Private* with Priming |  |  | 0.604  (0.504) | -0.024  (0.420) |  |  |  |  |
|  |  |  |  |  |  |  |  |  |
| Venmo-*Public* with Priming |  |  |  |  | 0.385  (0.315) | 0.271  (0.313) |  |  |
|  |  |  |  |  |  |  |  |  |
| Venmo-*Friends* with Priming |  |  |  |  |  |  | -0.267  (0.181) | -0.395^**^  (0.186) |
|  |  |  |  |  |  |  |  |  |
| Venmo Usage Controls | N | Y | N | Y | N | Y | N | Y |
| Item FE | Y | Y | Y | Y | Y | Y | Y | Y |
| Constant | 1.106^***^ | 1.130^***^ | 1.032^***^ | 1.348^*^ | 1.277^***^ | 1.493^**^ | 1.023^***^ | 0.648^*^ |
|  | (0.131) | (0.286) | (0.273) | (0.752) | (0.229) | (0.591) | (0.186) | (0.341) |
| Observations | 1640 | 1590 | 540 | 530 | 530 | 510 | 570 | 550 |
| R-Squared | 0.032 | 0.035 | 0.035 | 0.063 | 0.095 | 0.141 | 0.097 | 0.162 |

Standard errors in parentheses

^*^ *p* < 0.10, ^**^ *p* < 0.05, ^***^ *p* < 0.01
