# Supplementary figures and images for: Paying in public: Peer effects, impression management, and willingness to pay on digital payment platforms
Source: PLoS One. 2026 Jul 1;21(7):e0340550. doi: 10.1371/journal.pone.0340550 (PMC13322516; doi:10.1371/journal.pone.0340550)

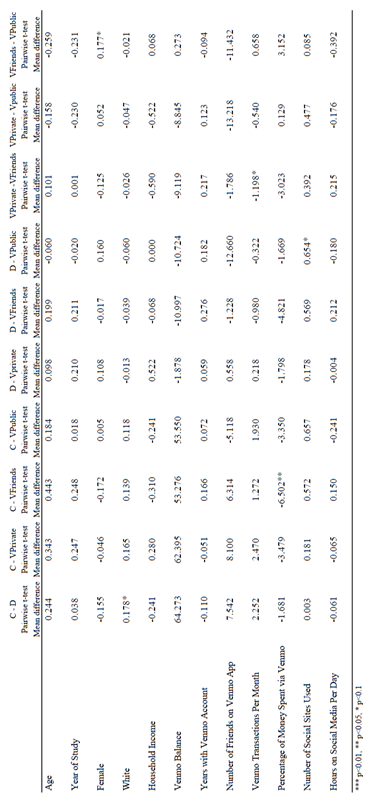

Supplement: S5 Table — This table reports pairwise t-tests of differences in mean participant characteristics in the cleaned sample. Pairwise differences which are statistically significantly different from zero are indicated by stars. “C” indicates the Credit treatment group, “D” represents the Debit treatment group, “VFriends” indicates the Venmo-Friends treatment group, “VPublic” indicates the Venmo-Public treatment group, “VPrivate” indicates the Venmo-Private treatment group. (TIF) [file pone.0340550.s005.tif]

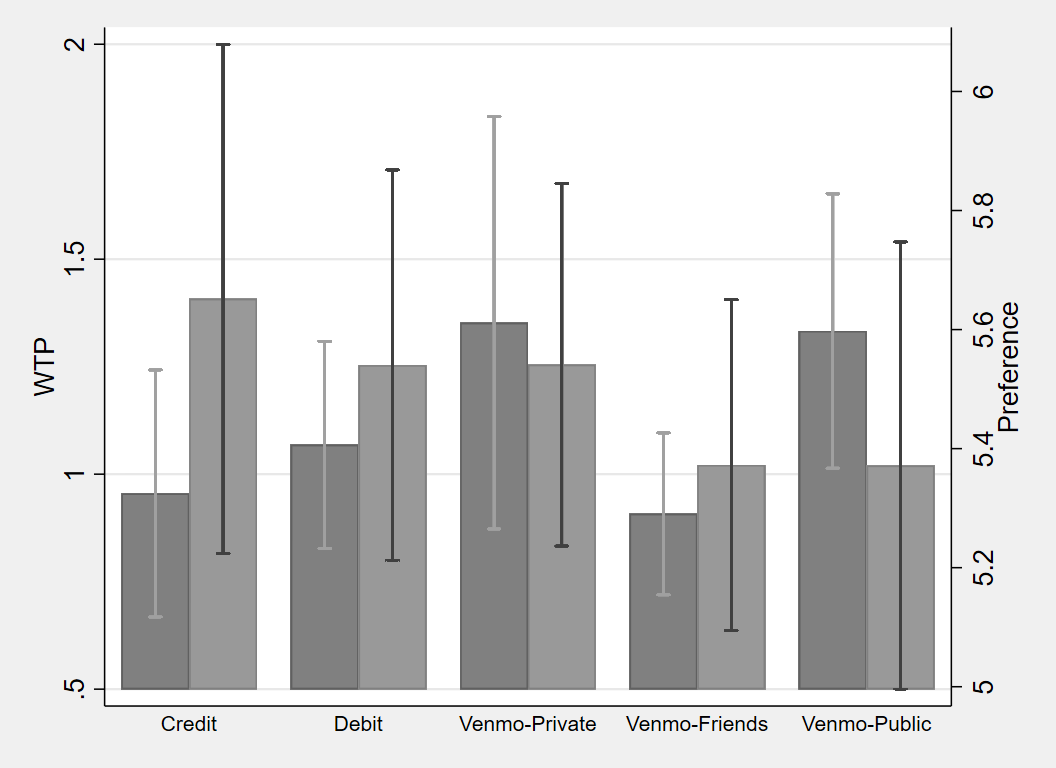

Supplement: S10 Fig — This figure illustrates mean willingness to pay (dark gray), and mean preference ratings (light gray) across all items for each treatment group. 95% confidence intervals are shown with standard errors clustered at the participant level. (TIF) [file pone.0340550.s010.tif]
