# Supplementary material for: Paying in public: Peer effects, impression management, and willingness to pay on digital payment platforms
Source: PLoS One. 2026 Jul 1;21(7):e0340550. doi: 10.1371/journal.pone.0340550 (PMC13322516; doi:10.1371/journal.pone.0340550)
Supplement: S6 Table — (DOCX) [file pone.0340550.s006.docx]

|  | (1) | (2) | (3) | (4) |
| --- | --- | --- | --- | --- |
|  | WTP-COVID | WTP-COVID | WTP-COVID | WTP-COVID |
| Credit Card | -0.231 | -0.179 | 0.498 | 0.515 |
|  | (0.526) | (0.677) | (0.822) | (0.976) |
|  |  |  |  |  |
| Venmo-Private | 1.728 | 1.543 | 2.423 | 2.233 |
|  | (2.038) | (1.821) | (2.347) | (2.128) |
|  |  |  |  |  |
| Venmo-Friends | -0.333 | -0.562 | -0.160 | -0.363 |
|  | (0.347) | (0.561) | (0.506) | (0.695) |
|  |  |  |  |  |
| Venmo-Public | 0.009 | -0.031 | 0.547 | 0.370 |
|  | (0.344) | (0.463) | (0.545) | (0.650) |
|  |  |  |  |  |
| Demographic Controls | N | N | Y | Y |
| Venmo Usage Controls | N | Y | N | Y |
| Item FE | Y | Y | Y | Y |
|  |  |  |  |  |
| Constant | 1.784^***^ | 1.980^***^ | 6.015 | 1.952 |
|  | (0.235) | (0.664) | (7.142) | (5.614) |
| Observations | 234 | 229 | 204 | 200 |
| R-Squared | 0.013 | 0.077 | 0.041 | 0.116 |
