# Supplementary material for: Paying in public: Peer effects, impression management, and willingness to pay on digital payment platforms
Source: PLoS One. 2026 Jul 1;21(7):e0340550. doi: 10.1371/journal.pone.0340550 (PMC13322516; doi:10.1371/journal.pone.0340550)
Supplement: S7 Table — (DOCX) [file pone.0340550.s007.docx]

|  | (1) | (2) | (3) | (4) |
| --- | --- | --- | --- | --- |
|  | WTP-Office | WTP-Office | WTP-Office | WTP-Office |
| Credit Card | -0.155 | -0.171 | -0.163 | -0.151 |
|  | (0.224) | (0.233) | (0.279) | (0.283) |
|  |  |  |  |  |
| Venmo-Private | 0.128 | 0.124 | 0.128 | 0.130 |
|  | (0.234) | (0.232) | (0.269) | (0.270) |
|  |  |  |  |  |
| Venmo-Friends | -0.152 | -0.184 | -0.214 | -0.191 |
|  | (0.193) | (0.192) | (0.210) | (0.209) |
|  |  |  |  |  |
| Venmo-Public | 0.303 | 0.173 | 0.296 | 0.155 |
|  | (0.265) | (0.258) | (0.286) | (0.282) |
|  |  |  |  |  |
| Demographic Controls | N | N | Y | Y |
| Venmo Usage Controls | N | Y | N | Y |
| Item FE | Y | Y | Y | Y |
|  |  |  |  |  |
| Constant | 1.182^***^ | 1.409^***^ | 1.782 | 1.207 |
|  | (0.166) | (0.299) | (1.559) | (1.872) |
| Observations | 702 | 687 | 612 | 600 |
| R-Squared | 0.039 | 0.056 | 0.059 | 0.071 |
