# Supplementary material for: Paying in public: Peer effects, impression management, and willingness to pay on digital payment platforms
Source: PLoS One. 2026 Jul 1;21(7):e0340550. doi: 10.1371/journal.pone.0340550 (PMC13322516; doi:10.1371/journal.pone.0340550)
Supplement: S8 Table — (DOCX) [file pone.0340550.s008.docx]

|  | (1) | (2) | (3) | (4) |
| --- | --- | --- | --- | --- |
|  | WTP-Toiletry | WTP-Toiletry | WTP-Toiletry | WTP-Toiletry |
| Credit Card | -0.218 | -0.191 | -0.150 | -0.128 |
|  | (0.240) | (0.248) | (0.287) | (0.297) |
|  |  |  |  |  |
| Venmo-Private | 0.129 | 0.111 | 0.213 | 0.170 |
|  | (0.244) | (0.248) | (0.262) | (0.269) |
|  |  |  |  |  |
| Venmo-Friends | -0.117 | -0.079 | -0.052 | -0.002 |
|  | (0.225) | (0.222) | (0.255) | (0.258) |
|  |  |  |  |  |
| Venmo-Public | 0.404 | 0.358 | 0.434 | 0.373 |
|  | (0.264) | (0.269) | (0.295) | (0.300) |
|  |  |  |  |  |
| Demographic Controls | N | N | Y | Y |
| Venmo Usage Controls | N | Y | N | Y |
| Item FE | Y | Y | Y | Y |
|  |  |  |  |  |
| Constant | 0.995^***^ | 0.948^***^ | 0.596 | 0.748 |
|  | (0.169) | (0.298) | (1.502) | (1.755) |
| Observations | 468 | 458 | 408 | 400 |
| R-Squared | 0.071 | 0.083 | 0.091 | 0.107 |
