# Supplementary material for: Paying in public: Peer effects, impression management, and willingness to pay on digital payment platforms
Source: PLoS One. 2026 Jul 1;21(7):e0340550. doi: 10.1371/journal.pone.0340550 (PMC13322516; doi:10.1371/journal.pone.0340550)
Supplement: S9 Table — (DOCX) [file pone.0340550.s009.docx]

|  | (1) | (2) | (3) | (4) |
| --- | --- | --- | --- | --- |
|  | WTP-Food | WTP-Food | WTP-Food | WTP-Food |
| Credit Card | 0.000 | 0.003 | 0.087 | 0.097 |
|  | (0.157) | (0.161) | (0.182) | (0.186) |
|  |  |  |  |  |
| Venmo-Private | 0.117 | 0.099 | 0.137 | 0.118 |
|  | (0.153) | (0.153) | (0.171) | (0.173) |
|  |  |  |  |  |
| Venmo-Friends | -0.145 | -0.144 | -0.191 | -0.161 |
|  | (0.136) | (0.137) | (0.150) | (0.152) |
|  |  |  |  |  |
| Venmo-Public | 0.231 | 0.169 | 0.235 | 0.180 |
|  | (0.168) | (0.153) | (0.192) | (0.173) |
|  |  |  |  |  |
| Demographic Controls | N | N | Y | Y |
| Venmo Usage Controls | N | Y | N | Y |
| Item FE | Y | Y | Y | Y |
|  |  |  |  |  |
| Constant | 0.530^***^ | 0.659^***^ | 1.659^**^ | 1.696 |
|  | (0.117) | (0.169) | (0.840) | (1.061) |
| Observations | 936 | 916 | 816 | 800 |
| R-Squared | 0.156 | 0.166 | 0.178 | 0.185 |
