# Supplementary material for: Paying in public: Peer effects, impression management, and willingness to pay on digital payment platforms
Source: PLoS One. 2026 Jul 1;21(7):e0340550. doi: 10.1371/journal.pone.0340550 (PMC13322516; doi:10.1371/journal.pone.0340550)
Supplement: S11 Table — (DOCX) [file pone.0340550.s011.docx]

|  | (1) | (2) | (3) | (4) |
| --- | --- | --- | --- | --- |
|  | Rating | Rating | Rating | Rating |
| Debit Card | -0.002 | 0.012 | -0.201 | -0.165 |
|  | (0.227) | (0.233) | (0.246) | (0.258) |
|  |  |  |  |  |
| Credit Card | 0.111 | 0.266 | -0.065 | 0.093 |
|  | (0.266) | (0.253) | (0.289) | (0.273) |
|  |  |  |  |  |
| Venmo - Friends | -0.169 | -0.106 | -0.225 | -0.143 |
|  | (0.209) | (0.222) | (0.212) | (0.231) |
|  |  |  |  |  |
| Venmo - Public | -0.169 | -0.227 | -0.379 | -0.441 |
|  | (0.245) | (0.247) | (0.265) | (0.273) |
|  |  |  |  |  |
| Demographic Controls | N | N | Y | Y |
| Venmo Usage Controls | N | Y | N | Y |
| Item FE | Y | Y | Y | Y |
|  |  |  |  |  |
| Constant | 5.862^***^ | 5.716^***^ | 8.202^***^ | 8.585^***^ |
|  | (0.188) | (0.328) | (1.380) | (1.576) |
| Observations | 2,337 | 2,287 | 2,040 | 2,000 |
| R-Squared | 0.043 | 0.053 | 0.043 | 0.055 |
