# Supplementary material for: Paying in public: Peer effects, impression management, and willingness to pay on digital payment platforms
Source: PLoS One. 2026 Jul 1;21(7):e0340550. doi: 10.1371/journal.pone.0340550 (PMC13322516; doi:10.1371/journal.pone.0340550)
Supplement: S12 Table — This table includes regression coefficients of estimations of WTP on payment form, item category, and their interaction. Robust standard errors, clustered at the participant level, are in brackets. *** p < 0.01, ** p < 0.05, * p < 0.1. (DOCX) [file pone.0340550.s012.docx]

|  | (1) | (2) | (3) |
| --- | --- | --- | --- |
| VARIABLES | WTP | WTP | WTP |
|  |  |  |  |
| *Categories (Food/Drinks omitted)* |  |  |  |
| Office Items | 0.175*** | 0.175*** | 0.181* |
|  | [0.054] | [0.054] | [0.100] |
| Toiletry Items | 0.504*** | 0.504*** | 0.490*** |
|  | [0.058] | [0.058] | [0.124] |
| COVID-Related Items | 1.180*** | 1.180*** | 0.965*** |
|  | [0.433] | [0.433] | [0.218] |
| *Treatment Groups (Debit omitted)* |  |  |  |
| Credit Card |  | -0.113 | 0.000473 |
|  |  | [0.190] | [0.158] |
| Venmo Private No Priming |  | -0.00967 | 0.0911 |
|  |  | [0.220] | [0.180] |
| Venmo Private Priming |  | 0.644 | 0.149 |
|  |  | [0.497] | [0.194] |
| Venmo Friends No Priming |  | -0.0714 | -0.0675 |
|  |  | [0.182] | [0.158] |
| Venmo Friends Priming |  | -0.277 | -0.246 |
|  |  | [0.178] | [0.153] |
| Venmo Public No Priming |  | 0.0420 | 0.0267 |
|  |  | [0.226] | [0.181] |
| Venmo Public Priming |  | 0.462* | 0.411* |
|  |  | [0.277] | [0.226] |
| *Interaction Terms* |  |  |  |
| Credit Card * Office |  |  | -0.156 |
|  |  |  | [0.170] |
| Venmo Private No Priming * Office |  |  | -0.0323 |
|  |  |  | [0.167] |
| Venmo Private Priming * Office |  |  | 0.0632 |
|  |  |  | [0.228] |
| Venmo Friends No Priming * Office |  |  | 0.0623 |
|  |  |  | [0.163] |
| Venmo Friends Priming * Office |  |  | -0.0973 |
|  |  |  | [0.181] |
| Venmo Public No Priming * Office |  |  | -0.0195 |
|  |  |  | [0.206] |
| Venmo Public Priming * Office |  |  | 0.154 |
|  |  |  | [0.251] |
| Credit Card * Toiletry |  |  | -0.218 |
|  |  |  | [0.186] |
| Venmo Private No Priming * Toiletry |  |  | -0.0149 |
|  |  |  | [0.195] |
| Venmo Private Priming * Toiletry |  |  | 0.0451 |
|  |  |  | [0.213] |
| Venmo Friends No Priming * Toiletry |  |  | -0.0631 |
|  |  |  | [0.186] |
| Venmo Friends Priming * Toiletry |  |  | 0.146 |
|  |  |  | [0.252] |
| Venmo Public No Priming * Toiletry |  |  | 0.132 |
|  |  |  | [0.216] |
| Venmo Public Priming * Toiletry |  |  | 0.211 |
|  |  |  | [0.250] |
| Credit Card * COVID |  |  | -0.231 |
|  |  |  | [0.476] |
| Venmo Private No Priming * COVID |  |  | -0.881*** |
|  |  |  | [0.280] |
| Venmo Private Priming * COVID |  |  | 4.669 |
|  |  |  | [4.382] |
| Venmo Friends No Priming * COVID |  |  | -0.0998 |
|  |  |  | [0.434] |
| Venmo Friends Priming * COVID |  |  | -0.303 |
|  |  |  | [0.342] |
| Venmo Public No Priming * COVID |  |  | -0.0520 |
|  |  |  | [0.359] |
| Venmo Public Priming * COVID |  |  | -0.372 |
|  |  |  | [0.355] |
| Constant | 0.859*** | 0.797*** | 0.819*** |
|  | [0.049] | [0.127] | [0.111] |
| Observations | 2,340 | 2,340 | 2,340 |
| R-squared | 0.021 | 0.032 | 0.063 |
